# Latency, thermal stability, and identification of an inhibitory compound of mirolysin, a secretory protease of the human periodontopathogen *Tannerella forsythia*

**DOI:** 10.1080/14756366.2021.1937619

**Published:** 2021-07-01

**Authors:** Krzysztof M. Zak, Mark J. Bostock, Irena Waligorska, Ida B. Thøgersen, Jan J. Enghild, Grzegorz M. Popowicz, Przemyslaw Grudnik, Jan Potempa, Miroslaw Ksiazek

**Affiliations:** a Helmholtz Zentrum München, Institute of Structural Biology, Neuherberg, Germany; b Malopolska Centre of Biotechnology, Jagiellonian University, Kraków, Poland; c Biomolecular NMR and Center for Integrated Protein Science Munich at Department Chemie, Technical University of Munich, Garching, Germany; d Department of Microbiology, Faculty of Biochemistry, Biophysics, and Biotechnology, Jagiellonian University, Krakow, Poland; e Department of Molecular Biology and Genetics, Aarhus University, Aarhus, Denmark; f Department of Oral Immunology and Infectious Diseases, University of Louisville School of Dentistry, Louisville, KY, USA

**Keywords:** Periodontitis, proteolysis, *Tannerella forsythia*, NMR-based fragment screening, protease inhibitors

## Abstract

Mirolysin is a secretory protease of *Tannerella forsythia*, a member of the dysbiotic oral microbiota responsible for periodontitis. In this study, we show that mirolysin latency is achieved by a “cysteine-switch” mechanism exerted by Cys23 in the N-terminal profragment. Mutation of Cys23 shortened the time needed for activation of the zymogen from several days to 5 min. The mutation also decreased the thermal stability and autoproteolysis resistance of promirolysin. Mature mirolysin is a thermophilic enzyme and shows optimal activity at 65 °C. Through NMR-based fragment screening, we identified a small molecule (compound (cpd) **9**) that blocks promirolysin maturation and functions as a competitive inhibitor (*K*_i_ = 3.2 µM), binding to the S1′ subsite of the substrate-binding pocket. Cpd **9** shows superior specificity and does not interact with other *T. forsythia* proteases or Lys/Arg-specific proteases.

## Introduction

Periodontitis is arguably the most prevalent infection-driven chronic inflammatory disease in humankind. It is estimated that up to 15% of adults worldwide experience severe forms of periodontitis^1^^,^^2^. In the United States alone, nearly 47% of people aged ≥30 years (approximately 65 million adults) suffer from this disease^3^. The progression of periodontitis is manifested by the destruction of tooth-supporting tissues. If left untreated, the disease can even lead to tooth loss^4^. Because of its chronic inflammatory and infectious character, periodontitis is associated with systemic diseases, such as rheumatoid arthritis, cardiovascular diseases, diabetes^5^, aspiration pneumonia^6^, and neurodegenerative diseases^7^.

A diverse dysbiotic community of microorganisms, forming the subgingival bacterial plaque^8^, is responsible for the development of periodontitis with *Porphyromonas gingivalis*, *Treponema denticola*, and *Tannerella forsythia*, playing a major role in disease progress^9^. Through the release of various virulence factors, including proteolytic enzymes^10^, these bacteria disturb homoeostasis in the periodontium, leading to a sustained host inflammatory response, which erodes tooth-supporting tissues^11^^,^^12^. The role of *P. gingivalis*^13^ and *T. denticola*^14^ secretory proteases in the pathobiology of periodontitis is very well characterised.

*T. forsythia* secretes a family of six multidomain proteases: three serine proteases (mirolase, miropsin-1, and miropsin-2) and three metalloproteases (karilysin, mirolysin, and forsilysin), referred to as KLIKK proteases. The proteases consist of a signal peptide (SP) responsible for promirolysin export to the periplasm, an N-terminal profragment (NTP) providing latency to the enzymes, a catalytic domain (CD), a C-terminal extension of unknown function, and a conserved C-terminal domain (CTD) serving as the signal for translocation across the outer membrane by the type IX secretion system (T9SS)^15^. By inactivating all complement pathways, mirolysin and karilysin synergistically protect the bacterium against the bactericidal activity of the complement system^16^.

Mirolysin, together with other M43B proteases, archetypal ulilysin and human pappalysins (pregnancy-associated plasma proteases) that hydrolyse growth hormone-binding proteins, shares unique specificity for Xaa–Arg and Xaa–Lys (P1–P1′) peptide bonds, and thus is dubbed LysargiNase^17^. In contrast to mirolase^18^ and karilysin^19^, the originally delineated NTP provided almost no latency to promirolysin^17^. However, the solved crystal structure of the mirolysin zymogen with the extended NTP revealed that promirolysin latency is exerted by Cys23 (cysteine-switch or Velcro mechanism)^20^.

Taking into account the ineffectiveness of current periodontitis therapies based on mechanical removal of the subgingival dental plaque combined with antibiotic treatment, specific inhibition of the activity of proteases secreted by major periodontal pathogens is considered a key target for the development of novel drugs^21^. So far, several inhibitors have been reported. These include Kyt-1 and Kyt-36^22^, N-terminal prodomain^23^, COR271, and COR286^7^, all targeting gingipains from *P. gingivalis*; the peptide SWFP, inhibiting karilysin from *T. forsythia*^24^; and doxycycline, inhibiting proteolytic activity of *T. denticola*^25^.

Fragment-based drug discovery provides a means of exploring chemical space using relatively small libraries of compounds (cpds), typically with <20 heavy atoms, with hits developed through fragment-growing or -linking approaches. In recent years, this has led to a number of compounds in the clinic or late-stage clinical trials^26^. Because of the relatively small size of libraries used (thousands of compounds), fragment-based drug discovery has become a standard method in academic drug discovery projects. Sensitive detection methods are required in the initial fragment screen, and NMR is commonly used because of its ability to detect weak binders (from low µM to mM range) and its lack of problems from target immobilisation during other biophysical experiments^27^. Consequently, NMR-based fragment screening provides a unique opportunity for finding novel small-molecule building blocks that can be utilised in the process of early-stage drug discovery projects.

Considering the crucial role of bacterial proteases in the aetiology of periodontitis, a significant health problem of humankind, it is important not only to characterise proteases in detail but also to develop novel compounds specifically targeting their activity. In this study, we demonstrate that Cys23 in the mirolysin NTP is responsible not only for latency but also for thermal and proteolytic stability of the zymogen. Furthermore, we characterised the extreme thermophilicity of mirolysin. Finally, employing NMR-based fragment screening, we identified a specific, competitive inhibitor, cpd **9**, which binds to the S1′ subsite in the mirolysin substrate-binding pocket.

## Materials and methods

### Chemicals, proteins, and reagents

*T. forsythia* KLIKK proteases (forsilysin, karilysin^15^, and mirolase^18^) and *P. gingivalis* gingipains (RgpB^28^ and Kgp^29^) were purified as described previously. Bovine trypsin, human thrombin, and plasmin were acquired from Merck Sigma-Aldrich (Darmstadt, Germany). The QuikChange Lightning Site-Directed Mutagenesis kit was purchased from Stratagene (La Jolla, CA, USA). Primers were synthesised by Genomed (Warsaw, Poland). Calbiochem Azocoll was purchased from Merck (Darmstadt, Germany). FTC-casein was acquired from Thermo Fisher Scientific (Waltham, MA, USA). All other chemical reagents were obtained from BioShop Canada (Burlington, ON, Canada).

### Protein expression and purification

Recombinant proteins were obtained by employing the *Escherichia coli* expression system, followed by affinity chromatography on glutathione Sepharose with on-column removal of the glutathione *S*-transferase (GST) tag by cleavage with PreScission protease, as described previously^17^. Homogeneous proteins were obtained by size-exclusion chromatography using a HiLoad 16/60 Superdex 75 pg column (GE Healthcare, Chicago, IL, USA) in 20 mM Tris, 0.02% NaN_3_, pH 8.0. The mature form of mirolysin was obtained by adding 10 mM CaCl_2_ to purified wild-type (wt) promirolysin (proM^wt^), followed by incubation for 2 weeks at 37 °C and dialysis against 5 mM Tris, 50 mM NaCl, 1 mM CaCl_2_, 0.02% NaN_3_, pH 8.0 to remove peptides resulting from mirolysin autoprocessing. Protein concentration was determined by measuring the absorbance at 280 nm, employing a NanoDrop spectrophotometer and the BCA assay (Sigma-Aldrich). All proteins were aliquoted immediately after purification and stored at −20 °C.

### ^15^N-labelling of proteins in* E. coli*

Isotopically labelled proteins were prepared by growing *E. coli* cells in ^15^N-labelled M9 mineral medium consisting of ^15^N-labelled M9 salts (33.7 mM Na_2_HPO_4_, 22 mM KH_2_PO_4_, 8.55 mM NaCl, 9.35 mM ^15^NH_4_Cl), 0.4% glucose, 1 mM MgSO_4_, 0.3 mM CaCl_2_, 1 µg/ml biotin and thiamine, and trace elements (134 µM EDTA, 31 µM FeCl_3_, 6.2 µM ZnCl_2_, 0.76 µM CuCl_2_, 0.42 µM CoCl_2_, 1.62 µM H_3_BO_3_, and 0.081 µM MnCl_2_). Recombinant mirolysin (^15^N-labelled) was purified as described above with an additional purification step after dialysis using a Superdex 75 10/300 GL column (GE Healthcare), pre-equilibrated with NMR buffer (5 mM Tris, 50 mM NaCl, 1 mM CaCl_2_, 0.02% NaN_3_, pH 8.0).

### Mutant construction

 lPromirolysin genes were previously cloned into pGEX-6P-1 vector encoding proteins proM^wt^ and proM^E225A^, in which the catalytic glutamic acid was replaced by alanine. Mutation of the Cys23 residue, responsible for mirolysin latency based on structural findings, into alanine and leucine was introduced by QuikChange Lightning Site-Directed Mutagenesis. Pairs of oligonucleotides used consisted of MK82 (GCCCCAGCGCACCGCCGGCTCTGAGTTG) and MK83 (CAACTCAGAGCCGGCGGTGCGCTGGGGC), and MK84 (GGGGCCCCAGCGCACCCTGGGCTCTGAGTTGAAT) and MK85 (ATTCAACTCAGAGCCCAGGGTGCGCTGGGGCCCC) for Cys23Ala and Cys23Leu mutations, respectively. Sequences of obtained genetic constructs were verified by DNA sequencing.

### Enzymatic activity assays

Mirolysin activity was routinely determined in assay buffer (50 mM Tris, 150 mM NaCl, 5 mM CaCl_2_, 0.02% NaN_3_, pH 7.5) supplemented with 0.05% Pluronic F-127, employing Azocoll or FTC-caseins as a substrate. Briefly, an Azocoll suspension was added to the sample in Eppendorf tubes and vigorously shaken for 15 min–2 h at 37 °C. Undigested Azocoll was removed by centrifugation (16,100 × *g*, 5 min, 4 °C), and the absorbance at 410 nm was measured using a Sunrise absorbance microplate reader (Tecan, Männedorf, Switzerland). In the case of FTC-casein, analysed samples were transferred into wells of a black microtiter plate (Nunc, Roskilde, Denmark), and then the FTC-casein solution was added. Proteolytic activity was recorded as an increase in fluorescence (excitation/emission = 485/538 nm) over time using a SpectraMax Gemini EM reader (Molecular Devices, San Jose, CA, USA).

### Effect of mutation on promirolysin

To determine the effect of mutation on the thermal stability of proM, differential scanning fluorimetry was performed using the Tycho NT.6 system (NanoTemper, Munich, Germany). Briefly, proM^E225A^, proM^E225A/C23A^, proM^E225A/C23L^, and mirolysin (8 µM) in 20 mM Tris, 5 mM CaCl_2_, 0.02% NaN_3_, pH 8.0 were transferred to glass capillaries, and unfolding profiles were obtained by measuring the fluorescence of intrinsic tryptophan and tyrosine residues detected at both 350 nm and 330 nm over a 30 °C/min temperature ramp in the range of 35–95 °C. The inflection temperature (*T*_i_), representing the unfolding transition, was determined by NanoTemper software. Additionally, the activities of proM^wt^, proM^C23A^, proM^C23L^ and mirolysin (10 nM) against FTC-casein (50 µg/ml) were determined.

### Thermal stability of mirolysin

Mirolysin (8 µM) was incubated in 20 mM Tris, 5 mM CaCl_2_, 0.02% NaN_3_, pH 8.0 at different temperatures (37–100 °C) for 30 min. Next, the activity of the 1 µl sample was measured in 200 µl, employing FTC-casein (50 µg/ml) as a substrate. In follow-up experiments, mirolysin was incubated at specific temperatures for 2 h. At specific time points, aliquots were withdrawn, and proteolytic activity was determined using FTC-casein as a substrate. Finally, the activity of mirolysin (50 nM) was measured at different temperatures ranging from 20 to 90 °C for 30 min using Azocoll (7.5 mg/ml) as a substrate.

### Gel electrophoresis

Mirolysin purification and processing were analysed by SDS–PAGE using 10% (T:C ratio, 33:1) gels and the Tris/Tricine buffer system^30^. Gels were stained with Coomassie Brilliant Blue G-250 in 10% acetic acid and destained subsequently in 30% methanol, 10% acetic acid; 10% acetic acid; and 1% acetic acid.

### Processing of promirolysin

For *in cis* autoprocessing, proM^wt^, proM^C23A^, and proM^C23L^ were prepared at a concentration of 1 mg/ml (∼28 µM) in assay buffer (20 mM Tris, 5 mM CaCl_2_, 0.02% NaN_3_, pH 8.0), and incubated for 360 h at 37 °C. At specific time points, aliquots were withdrawn for measurement of proteolytic activity against FTC-casein (50 µg/ml) and SDS–PAGE analysis of samples supplemented with 10 mM 1,10-phenanthroline, and were incubated for 5 min at room temperature before the addition of sample buffer. To investigate *in trans* activation of promirolysin, we monitored concentration- and time-dependent autoprocessing of different variants of the zymogen proM^E225A^. In the first case, proM^E225A^, proM^E225A/C23A^, and proM^E225A/C23L^ (1 mg/ml) were incubated with different concentrations of mirolysin at proM^E225A^/mirolysin molar ratios of 10, 100, and 1000 in assay buffer at 37 °C for 1 h. The enzymatic reaction was stopped by the addition of 10 mM 1,10-phenanthroline and incubation for 5 min at room temperature. Obtained samples were analysed by SDS–PAGE. In the time-dependent experiment, proM^E225A^, proM^E225A/C23A^, and proM^E225A/C23L^ (1 mg/ml) were incubated with mirolysin at a molar ratio of 400:1 in assay buffer at 37 °C for 288 h. At specific time points, aliquots were collected, the enzymatic reaction was stopped as described above, and samples were analysed by SDS–PAGE.

### NMR-based fragment screening and validation

The custom-selected Maybridge diversity library of 1500 fragments was screened using the saturation transfer difference (STD) ligand-based approach^31^, with fragments pooled into cocktails of five fragments each. STD spectra were recorded at a concentration of 10 µM mirolysin, with the final concentration of each fragment in the sample at 1 mM. D6-DMSO was used for the lock signal. STD experiments used alternating on- and off-resonance saturation (2s saturation, Gaussian pulse, +0.6 ppm, −5 ppm, 128 scans) with water suppression using a double-pulsed-field gradient spin-echo (DPFGSE) watergate W5 double-echo sequence^32^^,^^33^. Spectra were compared with a reference containing d6-DMSO only. Cocktails that showed a significant STD signal were identified, and the component fragments were rescreened individually (deconvolution screen). Hits identified using STD–NMR, based on the intensity ratio of ligand signals between the STD experiment and a 1D reference spectrum, were validated using ^1^H,^15^N heteronuclear single quantum coherence (HSQC) experiments with water flip back and watergate water suppression^34^^,^^35^, with spectra assessed for chemical shift perturbations (CSPs) relative to a control containing d6-DMSO. Compounds were screened at a 5:1 ligand:protein ratio, with protein concentration at ∼100 µM. Samples contained 10% D_2_O for locking. Spectra were recorded with 40 scans, and a ^1^H spectral width of 9615.4 Hz and a ^15^N spectral width of 2128.9 Hz, with 2048 and 256 complex points, respectively. All experiments were recorded at 298 K using a Bruker Avance 600 MHz spectrometer (^1^H frequency, 600 MHz) equipped with a 5 mm QCI cryoprobe. Titration experiments were carried out by adding increasing amounts of 100 mM stock solutions of cpd **9** or cpd **10** (dissolved in d6-DMSO) to ^15^N-labelled mirolysin (∼70-80 µM). Two-dimensional (2D) ^1^H-,^15^N-HSQC spectra were collected on a Bruker Avance 600 MHz spectrometer (^1^H frequency, 600 MHz, as above) (cpd **9**) at 298 K. Weight-averaged CSPs were calculated according to Equation (1):

(1) Δδ=(ΔδHN)2+(0.15 × ΔδN15)2

and CSPs were fit to the standard equation for the dissociation constant (*K*_D_) in fast exchange using OriginPro software^36^.

### Screening of the inhibitory activity of tested fragments

Mirolysin (40 nM) was mixed with each of the 21 compounds (2 µM) (Supplementary Table 1) in assay buffer (50 mM Tris, 150 mM NaCl, 5 mM CaCl_2_, 0.02% NaN_3_, 0.05% Pluronic F-127, pH 7.5) in a 96-well plate (total volume, 100 µl). After 15 min at 37 °C, 100 µl of the substrate solution FTC-casein (100 µg/ml) was added, and substrate hydrolysis was monitored. The percentage of inhibition was determined relative to the sample containing the enzyme without any compound added (control sample).

### N-terminal sequence analysis

Samples were separated by SDS–PAGE and electrotransferred in 10 mM *N*-cyclohexyl-3-aminopropanesulfonic acid, 10% methanol, pH 11 onto a polyvinylidene difluoride membrane using a mini trans-blot module (Bio-Rad, Hercules, CA, USA). Protein bands were visualised by Coomassie Brilliant Blue R-250 staining, excised, and analysed by automated Edman degradation using a Procise 494HT amino acid sequencer (Applied Biosystems, Carlsbad, CA, USA).

### Effect of compounds on autoprocessing

ProM^wt^ (70 µM) was activated at 37 °C in 5 mM Tris, 50 mM NaCl, 0.02% NaN_3_, pH 8.0, alone or in the presence of a 50 M excess of different compounds. At specific time points, aliquots were withdrawn and analysed by SDS–PAGE (10% gels; 4 µl sample, preincubated with 1:1 (vol:vol) of 20 mM 1,10-phenanthroline for 5 min at room temperature), and measured in a proteolytic activity assay, employing FTC-casein (25 µg/ml) as a substrate.

### Determination of *K*_D_ by microscale thermophoresis

The Monolith NT.115 instrument (NanoTemper) was used to analyse binding interactions between mirolysin and compounds **9**, **10**, and **21**. Lysine residues of mirolysin were fluorescently labelled with the Monolith NT Protein Labelling Kit RED-NHS-Red dye (NanoTemper), strictly according to the manufacturer’s instruction (a 7 M excess of dye was used). Compounds at concentrations ranging from 1.5 to 25 000 nM were incubated for 5 min at 20 °C with 20 nM mirolysin. Experiments were performed in 0.1 M Tris, 0.15 M NaCl, 5 mM CaCl_2_, 0.05% Pluronic-F127, 0.02% NaN_3_, pH 7.5. Samples were loaded into Monolith NT.115 Standard Treated glass capillaries (NanoTemper), and an initial fluorescence measurement followed by a thermophoresis measurement was carried out using 80% LED power and high microscale thermophoresis (MST) power. *K*_D_ values were calculated using MO. Affinity Analysis software (NanoTemper). Each experiment was performed in triplicate.

### Dose-dependent inhibition

Mirolysin (0.1 µM) was incubated with increasing concentrations of compounds (0–100 µM) in 50 µl assay buffer (50 mM Tris, 0.15 M NaCl, 5 mM CaCl_2_, 0.02% NaN_3_, pH 7.5) for 15 min at 37 °C, before 200 µl Azocoll suspension (15 mg/ml in assay buffer) was added.

### Screening of compound specificity

*T. forsythia* KLIKK proteases (mirolysin, mirolase, karilysin, and forsilysin); *P. gingivalis* gingipains (RgpB and Kgp), preactivated in gingipain assay buffer (GAB; 0.1 M Tris, 0.15 M NaCl, 5 mM CaCl_2_, 0.02% NaN_3_, pH 7.5, supplemented with 10 mM l-cysteine) for 15 min at 37 °C; human proteases (thrombin and plasmin); and bovine trypsin at a concentration of 100 nM were incubated with a 100 M excess of cpd **9** and cpd **10** in GAB or GAB supplemented with 0.05% Pluronic-F127 and 10 mM l-cysteine (gingipains only) for 15 min at 37 °C. The residual proteolytic activity was determined employing Azocoll (12 mg/ml) or, for gingipains only, FTC-casein (50 µg/ml), as described above.

### Fragment docking

Fragment docking was carried out using AutoDock 4.2 with AutoDockTools^37^. PRODRG was used to prepare molecular topologies of the input ligand files^38^. Docking was performed using a Lamarckian genetic algorithm with 2,500,000 energy evaluations per run and 1000 runs performed for each compound analysed.

### Determination of inhibition constant (K_i_)

Mirolysin (6 nM) was incubated at 37 °C in 0.1 M Tris, 0.15 M NaCl, 5 mM CaCl_2_, 0.05% Pluronic-F127, 0.02% NaN_3_, pH 7.5 in the presence of increasing concentrations of cpd **9** (0–7.2 µM) and cpd **10** (0–38.4 µM) in microtiter plate wells (total volume, 100 µl). After 15 min of incubation at 37 °C, 100 µl fluorogenic protein substrate FTC-casein was added at different concentrations (5–120 µg/ml), and the residual proteolytic activity was recorded. The mode of inhibition of target proteases by the compounds was determined graphically using the Lineweaver–Burk plot using Equation (2):

(2) $$\frac{1}{V}=\frac{K_{m}}{V_{\max}}\times \frac{1}{\left[S\right]}+\frac{1}{V_{\max}}$$

where *V* is the reaction velocity, *V*_max_ is the maximum *V*, *K*_m_ is the Michaelis–Menten constant, and [*S*] is the substrate concentration. The *K*_i_ was determined using a macro for competitive inhibition from GraphPad Prism software (La Jolla, CA, USA) and Equation (3):

(3) $$V=V_{\max}\times \frac{\left[S\right]}{K_{m}^{\mathrm{obs}}+[S]};\;K_{m}^{\mathrm{obs}}=K_{m}\times (1+\frac{\left[I\right]}{K_{i}})$$

where $K_{m}^{\mathrm{obs}}$ is the Michaelis–Menten constant in the presence of inhibitor, and [*I*] is the inhibitor concentration.

### Crystallisation of the mirolysin–cpd 9 complex

Mirolysin in 5 mM Tris, 50 mM NaCl, pH 8.0 was concentrated to 15 mg/ml and mixed with cpd **9** (final concentration, 10 mM). Screening for crystallisation conditions was performed using commercially available buffer sets (Molecular Dimensions, Sheffield, UK) in a sitting-drop vapour-diffusion setup by mixing 0.2 µl of a mirolysin–cpd **9** solution and 0.2 µl buffer solution. Crystals were obtained at room temperature from a solution containing 0.2 M zinc acetate dihydrate, 0.1 M sodium acetate, pH 4.5, and 10% (vol/vol) PEG 3000.

### Structure determination and refinement

Crystals were cryoprotected in 20% ethylene glycol in the mother liquor and flash-cooled in liquid nitrogen. Crystallographic experiments were performed on the X06DA beamline at the Swiss Light Source, Paul Scherrer Institut (Villigen, Switzerland). Data were indexed, integrated, and scaled using XDS^39^, and subsequently merged using Aimless^40^. The final structure was solved by molecular replacement using Phaser^41^, and the Protein Data Bank (PDB) 6R7W model was used as a search model^20^. The model was manually built using Coot^42^, and further refinement was carried out using REFMAC5^43^. Data-collection and -refinement statistics are presented in Supplementary Table 2. The final model was deposited in the PDB under accession number 7OD0.

## Results

### Mutant construction and protein purification

Under physiological conditions, hydrolysis of peptide bonds is irreversible; thus protease activity is a tightly controlled process to prevent any proteolytic damage. Such control is often achieved by the synthesis of proteolytic enzymes as inactive zymogens. Zymogenicity or latency is mainly exerted by the NTP. Despite possessing the NTP^15^, promirolysin had almost no latency at all, immediately processing itself into a mature form composed only of the CD^17^. Reanalysis of the mirolysin sequence (UniProt accession no. A0A0F7IPS1) with an updated tool predicting SP sequences^44^ revealed the presence of two possible SPs: one longer (M^1^–G^24^) SP and one SP shorter by five amino acids, M^1^–S^19^. Thus, the mirolysin NTP could be longer by five amino acids: Q^20^RTCG. Considering that the latency of metalloproteases is exerted by two main mechanisms, aspartate- and cysteine-switch mechanisms, in which catalytic zinc is chelated either by Asp or Cys, respectively, in the zymogen, an extension of the mirolysin NTP by a fragment containing Cys could affect the zymogenicity of this metalloprotease.

To verify this possibility, the recombinant NTP–CD (Q^20^–S^331^) variant of mirolysin, proM, was expressed, purified, and characterised. In stark contrast to a previous study^17^, the extended mirolysin zymogen proM was stable for a much longer time, in keeping with the crystal structure of promirolysin, revealing that Cys23 interacts with the catalytic zinc^20^. To experimentally verify the role of Cys23 in mirolysin latency, Cys23 was mutated into Ala and Leu in constructs encoding both wt proM (Q^20^–S^331^) and the corresponding catalytically inactive mutant, in which the catalytic residue Glu225 was replaced by Ala, proM^E225A^. Recombinant proteins were expressed as fusion proteins with an N-terminal GST tag and purified to homogeneity by affinity chromatography on glutathione Sepharose, with the GST tag removed by on-column cleavage with PreScission protease, followed by size-exclusion chromatography (data not shown).

### Effect of the Cys23 mutation on promirolysin activity and thermal stability

**Figure 1.**
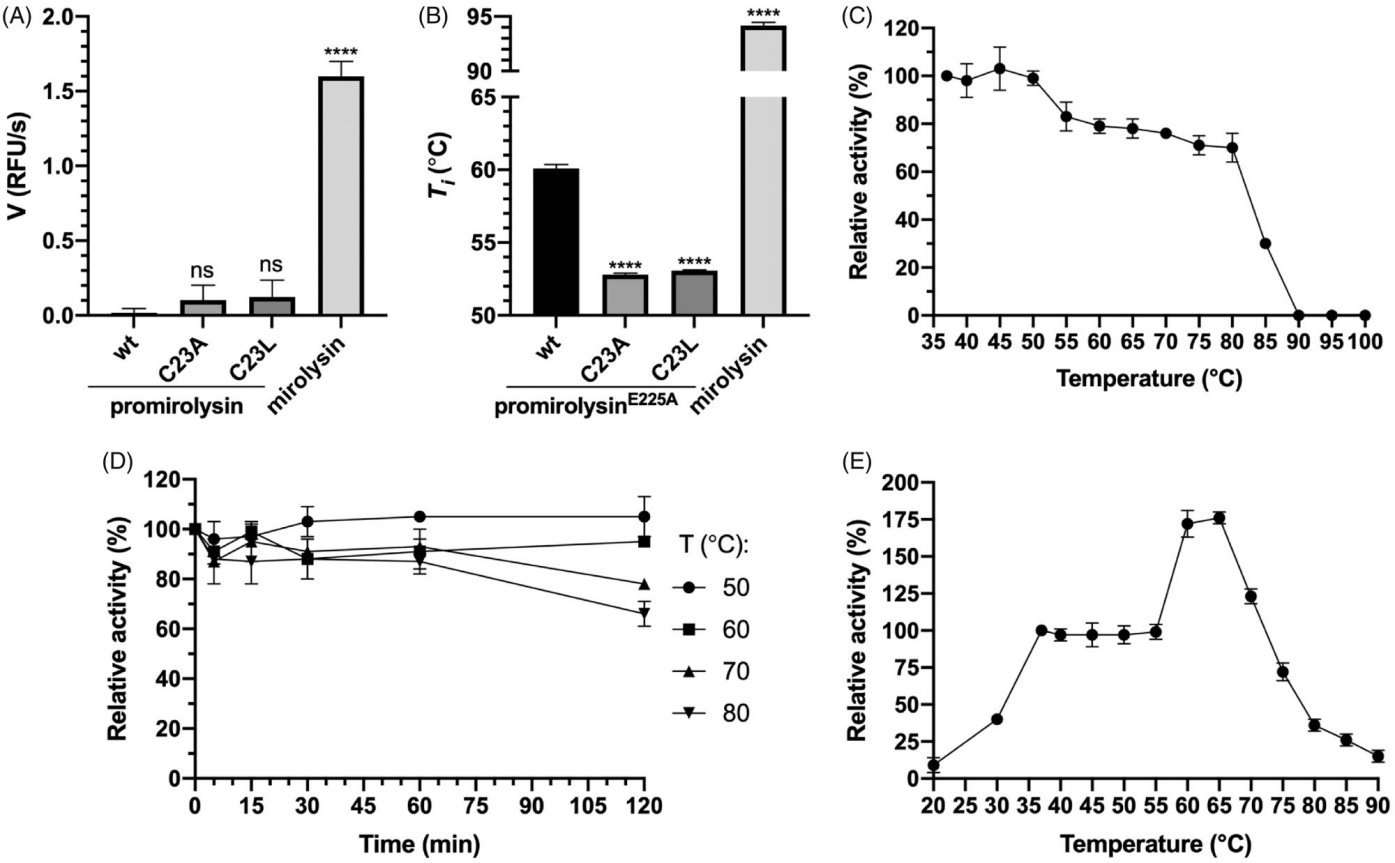
Enzymatic activity and stability. (A, B) The proteolytic activity (A) and the inflection temperature (*T*_i_) (B) of promirolysin (proM) and promirolysin^E225A^ (proM^E225A^), respectively: values for wild-type (wt) and mutant proteins C23A and C23L, and mirolysin, were determined by an activity assay employing FTC-casein as a substrate and differential scanning fluorimetry, respectively. The mean value ± SD resulting from triplicate measurements is shown. Statistical significance was determined by analysis of variance; ns, not significant; ****p< .0001. (C, D) Mirolysin was incubated at different temperatures for 30 min (C) or 2 h (D). During the longer incubation, aliquots were withdrawn at specific time points. After incubation, proteolytic activity in all samples was determined by employing FTC-casein as a substrate. (E) Activity of mirolysin against Azocoll was determined at the indicated temperatures after 30 min. The proteolytic activity at 37 °C (C, E) or at time 0 (D) was arbitrarily taken as 100%. Results presented are mean ± SD (*N* = 3).

To determine the effect of Cys23 on promirolysin latency and thermal stability, we measured the proteolytic activity of proM^wt^, proM^C23A^, and proM^C23L^, and compared it with that of mirolysin. proM^wt^ was not active at all, while, in the case of proM^C23A^ and proM^C23L^, low activity was detected, but it was almost 15 times lower than that for mirolysin (Figure 1(A)). These findings suggest that proM^C23A^ and proM^C23L^ can undergo at least partial activation. We also checked the effect of both mutations on the thermal stability of proM by differential scanning fluorimetry. In this experiment, we used proM^E225A^ as the reference wt protein to exclude the possibility of autoproteolysis during heating of the sample. Both mutations decreased the inflection temperature (*T*_i_) of proM^E225A^ by 7 °C. The *T*_i_ of mirolysin was almost 35 °C higher than that of proM^E225A^ (Figure 1(B)).

### Analysis of the thermal stability of mirolysin

To further investigate the thermal stability of mirolysin, the enzyme was incubated at different temperatures for 30 min, and the proteolytic activity was determined. The full activity was observed up to 50 °C, and then it gradually decreased by 20% from 50 °C to 80 °C, and dropped precipitously to zero at 90 °C (Figure 1(C)). Subsequently, mirolysin was kept at four temperatures: 50, 60, 70, and 80 °C for 2 h, after which the proteolytic activity was monitored. After an initial slight decrease, the activity remained stable for 1 h at all temperatures. For the two highest temperatures, a significant decrease in activity did not occur until 2 h (Figure 1(D)). Finally, the proteolytic activity of mirolysin against Azocoll was determined at different temperatures ranging from 20 to 90 °C. Compared with mirolysin activity at a temperature of 37 °C, the activity of mirolysin at 20 °C was more than ten times lower, rising to 40% at 30 °C. In the temperature range of 40–55 °C, activity was stable, increasing drastically thereafter and peaking at 175% at 65 °C. After this, a gradual decline in activity began, but mirolysin retained 75% of its activity even at 75 °C (Figure 1(E)). Mirolysin showed notable thermophilicity, reaching its highest activity at 65 °C.

#### Promirolysin activation *in cis*

**Figure 2.**
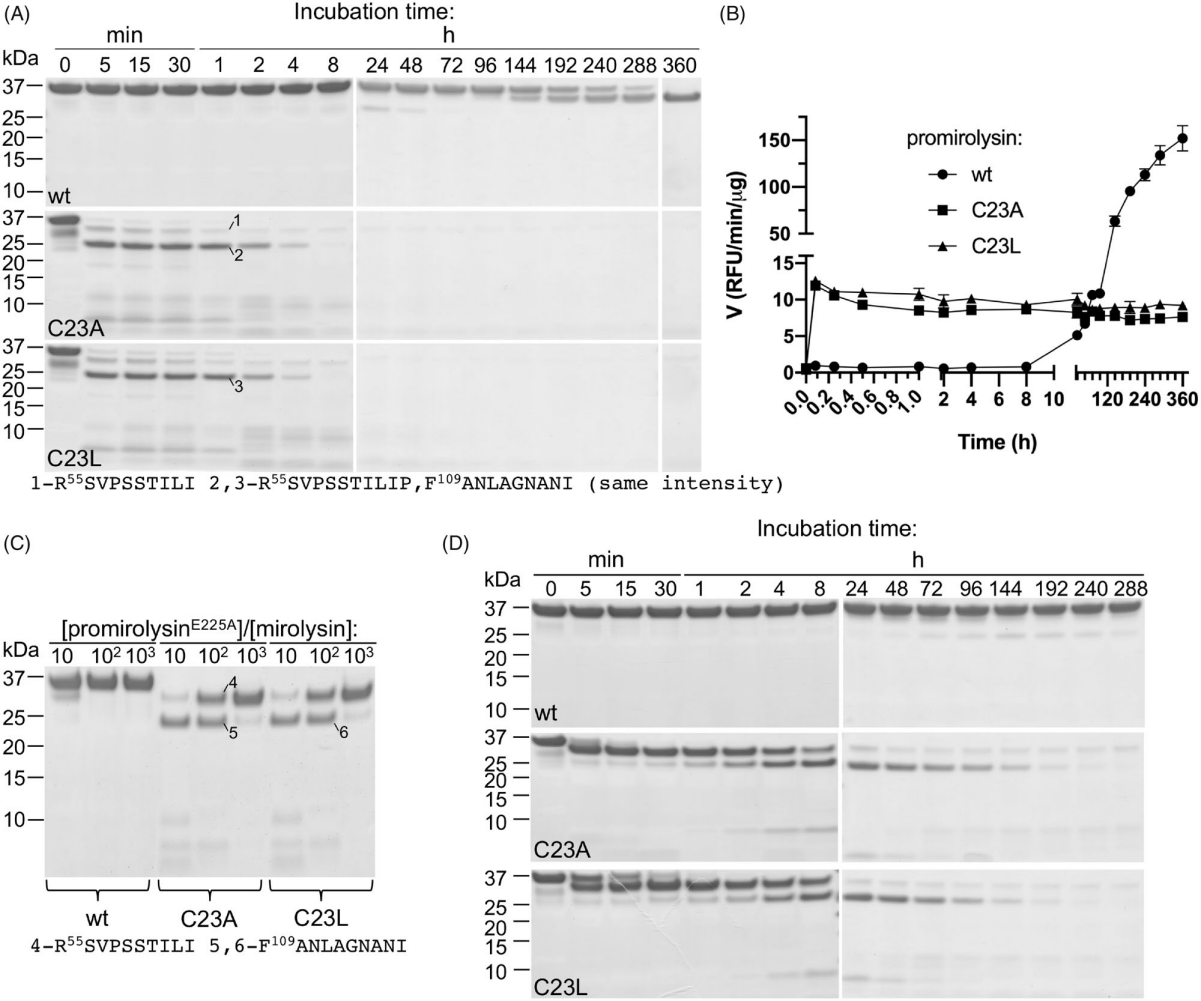
Activation *in cis* (autoprocessing) (A, B) and *in trans* (C, D) of promirolysin (proM). (A, B) For proM, wild-type (wt), C23A, and C23L proteins were incubated at 37 °C for 360 h. At specific time points, aliquots were withdrawn and analysed by SDS–PAGE (A), and proteolytic activity was measured by employing FTC-casein as a substrate (B). Data shown are mean ± SD (*N* = 3). (C, D) For proM^E225A^, wt, C23A, and C23L proteins were incubated at 37 °C with mirolysin at three different molar ratios: 10:1; 100:1, and 1000:1 for 1 h (C), or at one molar ratio (400:1) for 288 h, and aliquots were withdrawn at specific time points. All collected samples were analysed by SDS–PAGE. Selected bands were excised and subjected to N-terminal sequencing, which allowed identification of the cleavage sites during activation of proM.

To characterise mirolysin autoprocessing (activation *in cis*) in detail, proM^wt^, proM^C23A^, and proM^C23L^ were incubated at 37 °C for 360 h, during which time aliquots were taken at specific time points for SDS–PAGE analysis and measurement of proteolytic activity employing FTC-casein (Figure 2(A,B)). In the case of proM^wt^, a transition of the 35 kDa zymogen into a 31 kDa mature enzyme, mirolysin, through cleavage at the S^54^R peptide bond, was observed as determined by N-terminal sequence analysis (Figure 2(A)). In total, 360 h were required to complete the activation (Figure 2(A)). The changes observed on gels were consistent with the changes in measured proteolytic activity. A significant increase in activity occurred after 144 h, when a clear band corresponding to the 31 kDa form could be observed (Figure 2(B)). The earlier increase in activity can be explained by the greater sensitivity of the FTC-casein assay than that of protein-band staining in SDS–PAGE. In the case of proM^C23A^ and proM^C23L^, changes in band patterns occurred much faster. Similar to proM^wt^, cleavage occurred first at the S^54^R position, but only 5 min were required to complete full processing of proM. Next, the 31 kDa form was further cleaved, first through proteolysis at the A^108^F peptide bond into the 25 kDa form, followed by almost complete degradation, first into fragments around 10 kDa and later into small peptides not detected on gels. After 360 h, only a weak band of the 31 kDa form was present in the sample (Figure (2A)). Rapid activation of both mutant proteins was accompanied by a burst in proteolytic activity, which remained at the same level during the whole incubation period (Figure (2B)). The 15-fold difference in activity between proM^wt^, and proM^C23A^ and proM^C23L^, correlated very well with the differences in the intensity of the bands, which exuded the 31 kDa form at the end of the experiment (Figure 2(A,B)). From this, it can be calculated that only ∼7% of both mutant proteins are processed to the stable and active 31 kDa form.

#### Promirolysin activation *in trans*

We also analysed activation *in trans*, during which the catalytically inactive zymogen of mirolysin (proM^E225A^) was subjected to proteolysis by mirolysin (Figure 2(C,D)). In the first concentration-dependent experiment, proM^E225A^ and two mutants, proM^E225A/C23A^ and proM^E225A/C23L^, were incubated for 1 h with increasing concentrations of mirolysin (Figure 2(C)). While proM^E225A^ remained intact with only a small amount of protein cleaved at a molar ratio of 10:1 (proM:mirolysin), both proM^E225A/C23A^ and proM^E225A/C23L^ were processed into a 31 kDa form even at the lowest enzyme concentration (1000:1), through cleavage at the S^54^R peptide bond. At a 10:1 molar ratio, the second cleavage at A^108^F was almost fully completed, resulting in degradation products around 10 kDa (Figure 2(C)). Next, time-dependent activation was performed, when proM^E225A^, proM^E225A/C23A^, and proM^E225A/C23L^ were incubated with mirolysin at a molar ratio of 400:1 for 288 h, and aliquots from different time points were analysed by SDS–PAGE (Figure 2(D)). In stark contrast to the wt zymogen proM^E225A^, for which no visible sign of transition into the 31 kDa form consisting only of the CD was detected, proM^E225A/C23A^ and proM^E225A/C23L^ were processed similarly as for activation *in cis*: first into a 31 kDa form through cleavage at the S^54^R peptide bond, followed by proteolysis at the A^108^F bond into a 25 kDa form. Subsequent degradation, first into fragments around ∼10 kDa and finally into small peptides not visible in gels, occurred. At the end of the incubation period, only a small amount of the 31 kDa form was present. Of note, proM^E225A/C23L^ is processed slower than proM^E225A/C23A^ for the first 8 h required to complete the first two cleavages at S^54^R and A^108^F, and then the activation rate of proM^E225A/C23A^ and proM^E225A/C23L^ is the same (Figure 2(D)).

### Fragment screening discloses a set of positively charged fragments interacting with mirolysin

**Figure 3.**
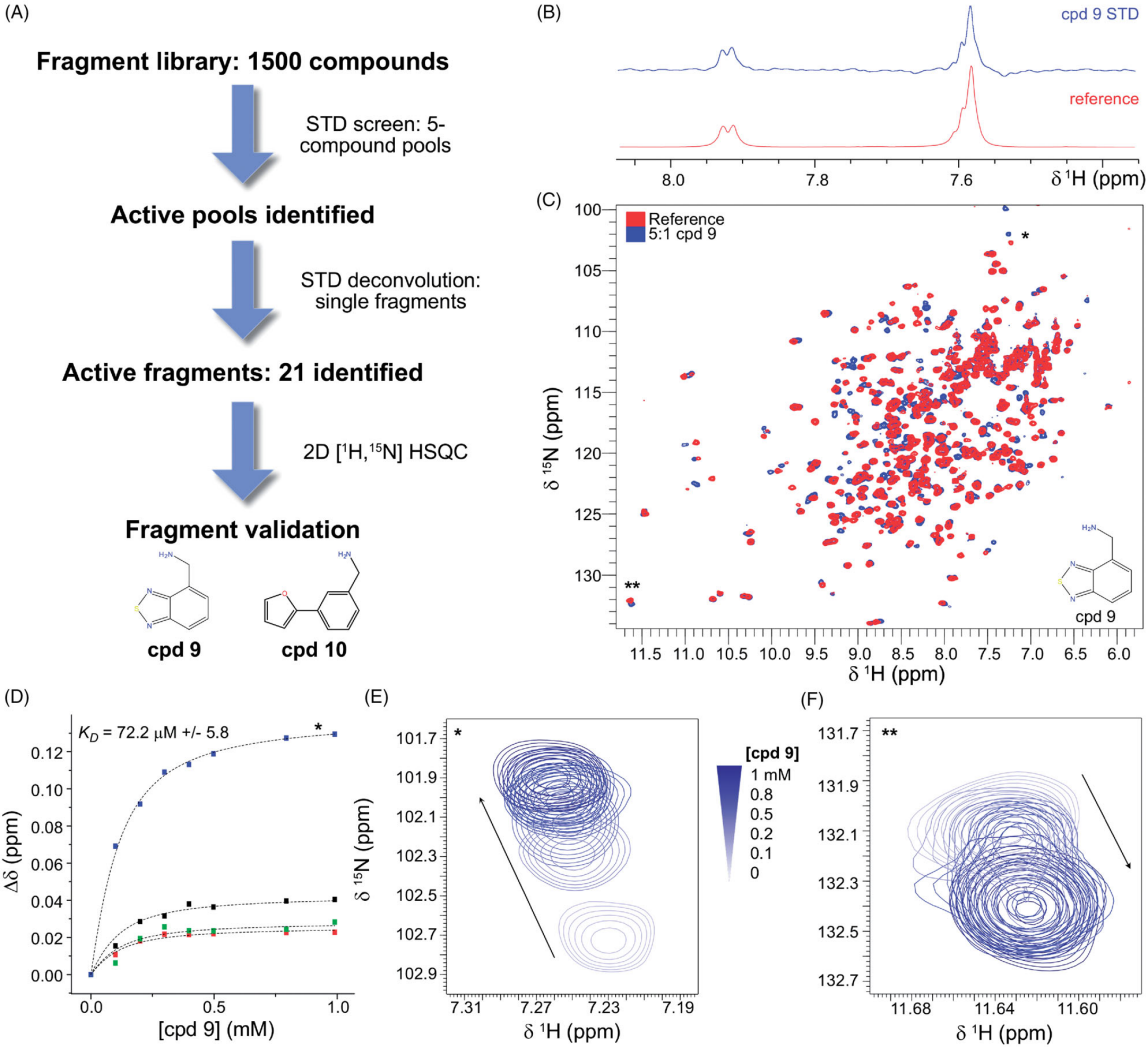
NMR-based fragment screening of mirolysin. (A) A summary of the fragment-screening process is shown. Initially, 10 μM mirolysin was screened against a fragment library of 1500 compounds in pools of five, using the STD experiment. Hits were identified and ranked according to the intensity of the STD signals. Subsequently, the constituent compounds of the active pools were screened individually, and the 21 compounds showing the largest STD signals were selected. After showing that some compounds had inhibitory properties against mirolysin, they were validated using 2D [^1^H,^15^N]-heteronuclear single quantum coherence (HSQC) experiments, with compounds added to ^15^N-labelled mirolysin at a 5:1 ratio. (B) The amide region of an STD experiment for active fragment cpd **9**, showing equivalent signals in the 1D reference experiment and the STD experiment. (C) 2D [^1^H,^15^N]-HSQC experiment for ^15^N-labelled mirolysin with cpd **9** added at a 5:1 ratio (blue), compared with a reference spectrum. (D) Titration of cpd **9** against ^15^N-labelled mirolysin, observed using 2D [^1^H,^15^N]-HSQC experiments and cpd **9** concentrations in the range of 0–1 mM. Several peaks were observed in fast exchange (E) and could be fitted to extract an approximate *K*_D_. Many peaks in the titration experiment were observed to be in slow exchange, for example, the peak shown in (F). The peaks shown in (E) and (F) are indicated on the full 2D spectrum in (C) by * and **. An overlay of the titration spectra is shown in Supplementary Figure 3. NMR spectra were recorded at 298 K on a Bruker Avance 600 MHz magnet (^1^H frequency, 600 MHz).

We screened a custom library provided by Maybridge of 1500 fragments using the STD ligand-based method^31^ (Figure 3(A)). STD spectra assess compound binding and release from a protein target, with magnetisation transfer from the protein to the ligand occurring on binding^45^. By comparing STD with one-dimensional (1D) spectra of the ligand–protein mixtures, we identified ligand signals present in the STD spectra that indicated binding. Compounds were initially screened in pools of five. For compound cocktail spectra indicating binding, deconvolution of cocktails was performed, and the contained fragments were rescreened using the STD method individually. Based on the ratio of intensities of the ligand signals between the STD and 1D spectra in the deconvolution screen, we subselected 21 compounds for further steps (screening round I) (Figure 3(B)). Despite the diverse chemical structures, almost all of the 21 identified fragments contained amine groups (except cpds **4**, **11**, and **14**) (Supplementary Table 1).

To check if any of these 21 compounds could inhibit mirolysin, they were mixed separately with the protease at a molar ratio of 50:1 (compound:enzyme), and residual proteolytic activity was determined by employing FTC-casein as a substrate (screening round II). Ten compounds (cpds **1**, **3**, **7**, **13**, **15**–**21**) reduced mirolysin activity by <10%, six compounds (cpds **2**, **4**, **9**–**11**, **14**) reduced mirolysin activity by 10–20%, and three compounds (cpds **5**, **6**, **12**) reduced mirolysin activity by 20–25%. For the remaining two compounds, **8** and **21**, no inhibition was observed (Supplementary Figure 1). The results did not show a clear correlation between the chemical structure and inhibitory properties of compounds.

Hits from screening round I were re-evaluated using ^1^H,^15^N HSQC spectra (screening round III), and analysed for CSPs to provide direct, protein-based evidence for fragment–protein interaction (Supplementary Table 1, Figure 3(C)). Spectra were ranked qualitatively on a scale from 0 to 5, based on the number and magnitude of the CSPs. Of 21 tested molecules, 18 showed no or very weak interaction, and three resulted in more significant changes in the recorded spectra (cpds **9**, **10**, and **17**) (Supplementary Figure 2). Compound formulas and ranking based on 2D HSQC are shown in Supplementary Table 1. In addition, to provide a more quantitative ranking of the 2D HSQC spectra, the CSP analyser tool^46^, which uses a machine-learning algorithm to assess CSPs in 2D screening, was used. Because of the crowded centre of the HSQC spectrum, where it is challenging for the algorithm to assess CSPs, as well as a large number of asparagine- and glutamine-side chain signals, a cut-off of 8.3 ppm was used in the F2 dimension. Cpd **17** was indicated as a hit, while cpds **9** and **10** were assigned a non-zero hit probability. All other compounds were assigned an activity probability of 0, consistent with our qualitative analysis. The three fragments showing the largest number of NMR spectral changes in HSQC screening (cpds **9**, **10**, and **17**) all contain primary amines, although, clearly, the complex character of fragment recognition spans beyond charged interactions. To conclude, the screening analysis revealed two compounds, **9** and **10**, which were positive hits in all three screening rounds.

Consequently, the interactions of cpds **9** and **10** with mirolysin were assessed by 2D NMR titration experiments (Figure 3(D–F)), Supplementary Figures 3–5). Titration with cpd **9** against mirolysin showed that many residues were in slow exchange, typically indicative of tighter binding (Figure 3(F), Supplementary Figure 3). Other residues were observed in fast exchange (Figure 3(E)), and were used to estimate the *K*_D_, giving a value of 72.2 µM (Figure 3D). For compound 10, titration data indicated that most residues with CSPs were in fast exchange (Supplementary Figures 4,5), with an estimated *K*_D_ of 278 µM, indicating significantly weaker binding. These values are typical for fragments that require further growing and/or medicinal chemistry optimisation to achieve clinically relevant potencies.

#### Inhibition of promirolysin activation *in cis*

**Figure 4.**
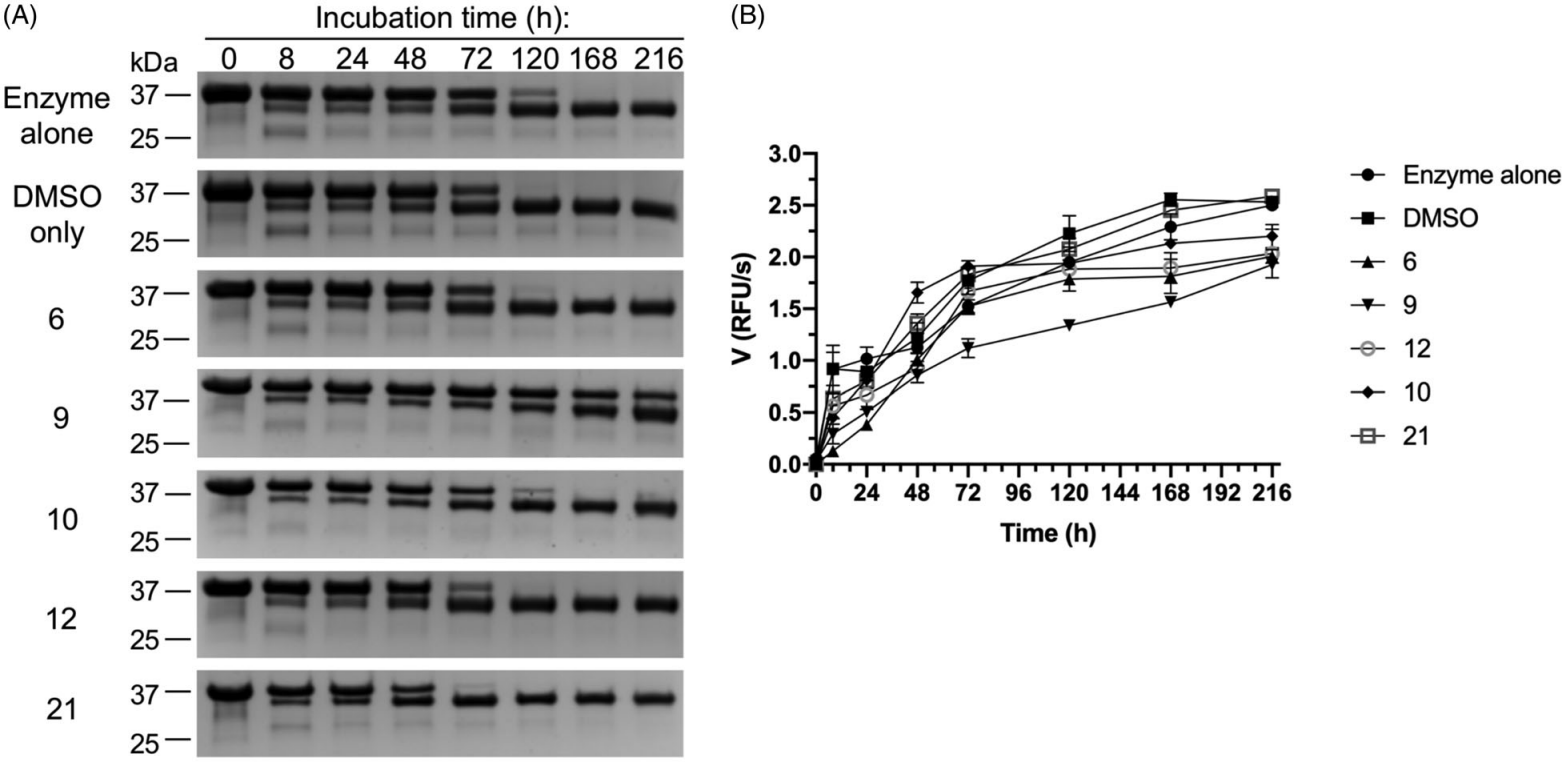
Effect of selected compounds on activation of promirolysin (proM). Wild-type proM (proM^wt^) was incubated at 37 °C alone and with DMSO (negative control) or a 50 M excess of compounds **6**, **9**, **10**, **12**, and **21**. At specific time points, aliquots were taken and analysed by SDS–PAGE (A), followed by measurement of proteolytic activity against FTC-casein as a substrate (B). Data shown are mean ± SD (*N* = 3).

As mentioned in the above sections, proM^wt^ undergoes autocleavage for full maturation of the zymogen (activation *in cis*) (Figure 2(A,B)). Thus, we assessed the effect of selected compounds identified in previous screening rounds, cpd **9** and cpd **10**, which were positive in all three screening rounds. Cpd **17**, identified as active by the CSP analyser, and giving significant CSPs (qualitative observation) was excluded because of lack of significant inhibition in screening round II. Cpds **6** and **12** were selected because they showed the most significant inhibitory activity in the proteolytic cleavage assay (screening round II) (Supplementary Figure 1). Cpd **21** (identified in screening round I) was included as a negative control (NC) for proM^wt^ activation *in cis* because it showed no inhibitory activity (screening round II) (Supplementary Figure 1). proM^wt^ was incubated alone or in the presence of a 50 M excess of compound at 37 °C for 216 h. At specific time points, aliquots were withdrawn and analysed by SDS–PAGE (Figure 4(A)), and measurements of proteolytic activity against FTC-casein were made (Figure 4(B)). Almost all of the compounds tested had no effect on proM^wt^ maturation, and the entire process was completed within 120 h. By contrast, cpd **9** significantly slowed proM^wt^ maturation from the 35 kDa zymogen to a 31 kDa mature form, and the whole process was not completed even after 216 h (Figure 4(A). Results from SDS–PAGE analysis were fully confirmed by measuring activity against FTC-casein. Only cpd **9** significantly decreased the activity of mirolysin in all analysed aliquots. After 216 h, the activity of mirolysin was reduced by 30%, not only by cpd **9**, but also by cpds **6** and **12**. Only cpd **21** and DMSO did not affect the proteolytic activity of mirolysin. These results corroborated the previous results of the inhibitory activity of 21 compounds from screening round II (Supplementary Figure 1).

### Interaction of cpd 9 and cpd 10 with mirolysin

**Figure 5.**
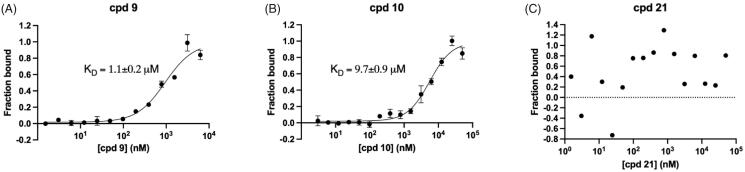
Determination of the dissociation constant (*K*_D_) by microscale thermophoresis. Fluorescently labelled mirolysin was titrated with increasing concentration of cpd **9** (A), cpd **10** (B), and cpd **21**, serving as a negative control (C). Binding data were fitted to determine *K*_D_ values. Results are presented as mean ± SD of three experiments.

**Figure 6.**
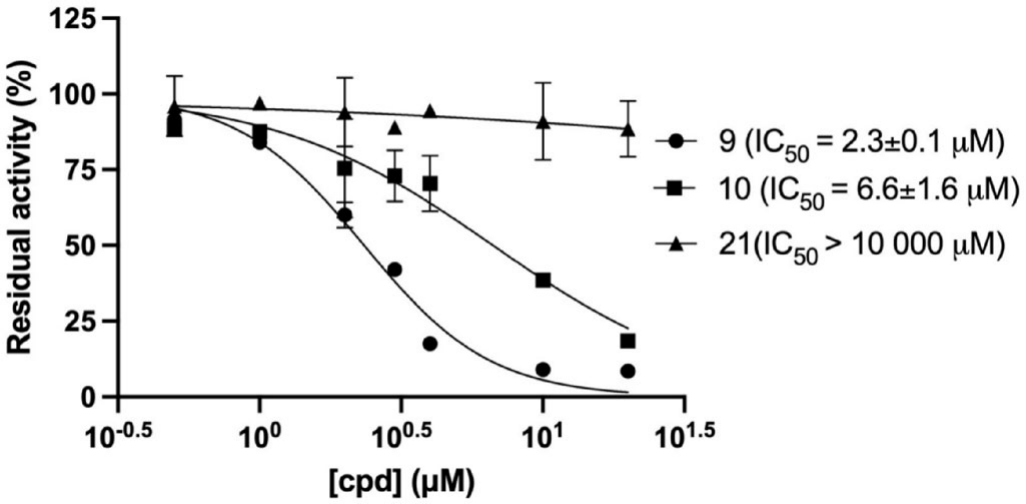
Dose-dependent inhibition of mirolysin by compounds **9**, **10**, and **21**. Mirolysin was preincubated with increasing concentrations of compounds before the residual activity was determined by employing Azocoll as a substrate.

We then quantitatively analysed the interaction of mirolysin with cpd **9** and cpd **10**, which were positive in all three screening rounds, by MST. The experiments were performed with fluorescently labelled mirolysin and serial dilutions of each compound. The *K*_D_ of the mirolysin–cpd interaction was 1.1 and 9.7 µM for cpd **9** and cpd **10**, respectively. Of note, in the case of cpd **21**, used previously as an NC, no interaction was observed (Figure 5(A–C)). In a dose-dependent experiment, mirolysin was inhibited with a half-maximal inhibitory concentration (IC_50_) of 2.3 and 6.6 µM by cpd **9** and cpd **10**, respectively. In the case of the NC, cpd **21**, almost no effect on mirolysin activity was observed (IC_50_ > 10,000 µM) (Figure 6). These results corroborated findings from inhibition of promirolysin autoactivation and, altogether, strongly indicated that cpd **9** is a potent inhibitor of mirolysin, identified from the analysed library of compounds.

#### Determination of inhibition constant (*K*_i_)

**Figure 7.**
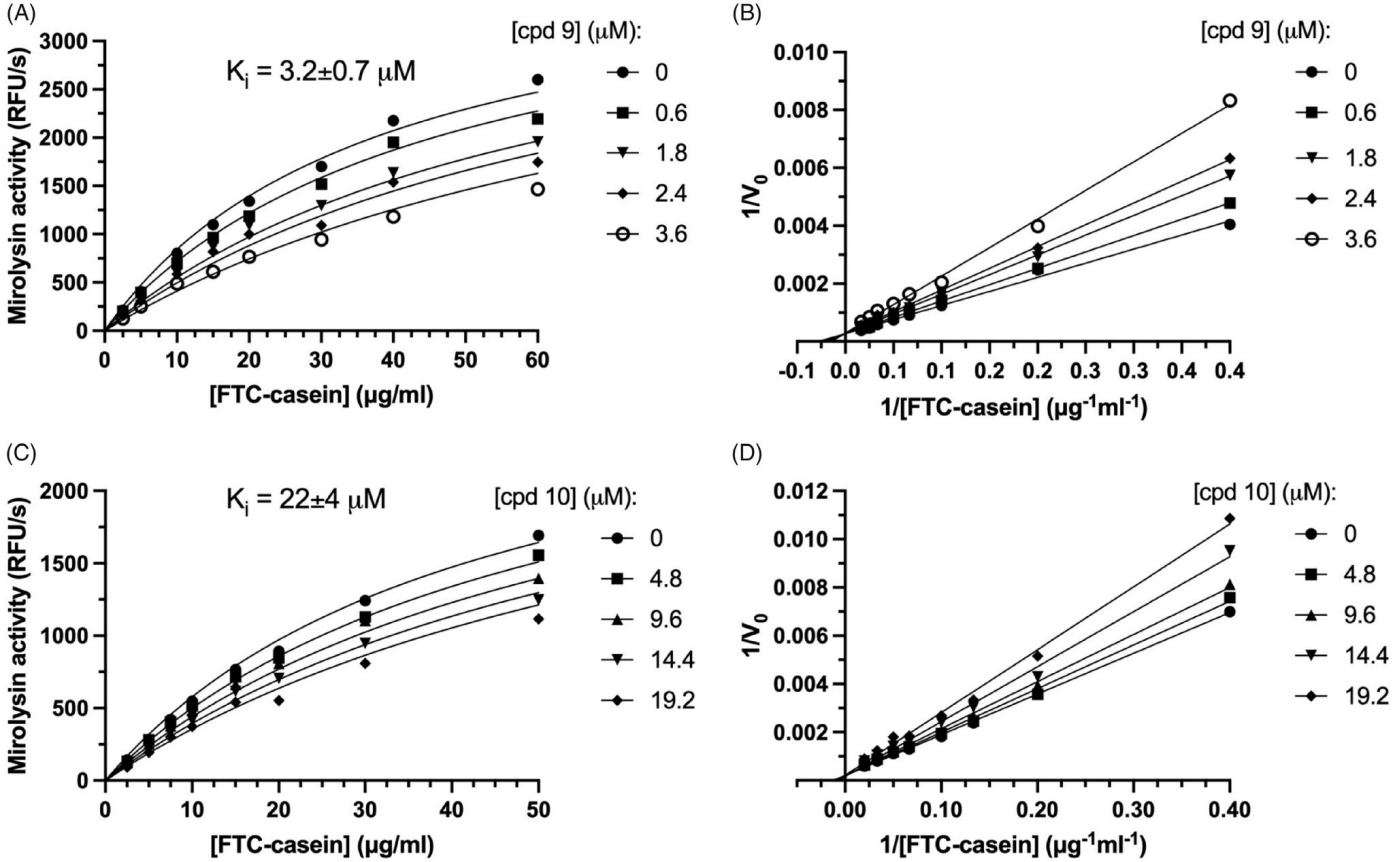
Determination of the inhibition constant (*K*_i_). Mirolysin was incubated with increasing concentrations of the appropriate compound for 15 min, and velocities of the reaction (*V*) were determined using different concentrations of FTC-casein as a substrate. Based on obtained raw data (A,C), Lineweaver–Burk plots were also prepared (reciprocal of *V* versus the reciprocal of substrate concentration) (B,D), which were used to determine the inhibition mechanism. *K*_i_ values were obtained using a macro for competitive inhibition from GraphPad Prism and raw data (A,C). Data are presented as mean ± SD (*N* = 3).

To further compare the inhibitory potency of cpd **9** and cpd **10**, and determine a mode of inhibition, kinetic analysis of inhibition was performed (Figure 7(A,C)). Inhibition followed Michaelis–Menten kinetics and was dependent on both substrate, FTC-casein, and inhibitor concentrations (cpd **9** and cpd **10**). To determine the mode of inhibition, a Michaelis–Menten analysis of substrate proteolysis was carried out (Figure 7 (B,D)). In both cases, linear curves fitted to the experimental points crossed at one point lying on the *y*-axis. Together, these findings show that both compounds were reversible competitive inhibitors of the target proteases. Based on this, we determined the *K*_i_, the best parameter describing potency of reversible inhibitors (Figure 7 (A,C)). Cpd **9** and cpd **10** inhibited mirolysin with a *K*_i_ of 3.2 and 22 µM, respectively.

### Analysis of compounds’ inhibition specificity

**Figure 8.**
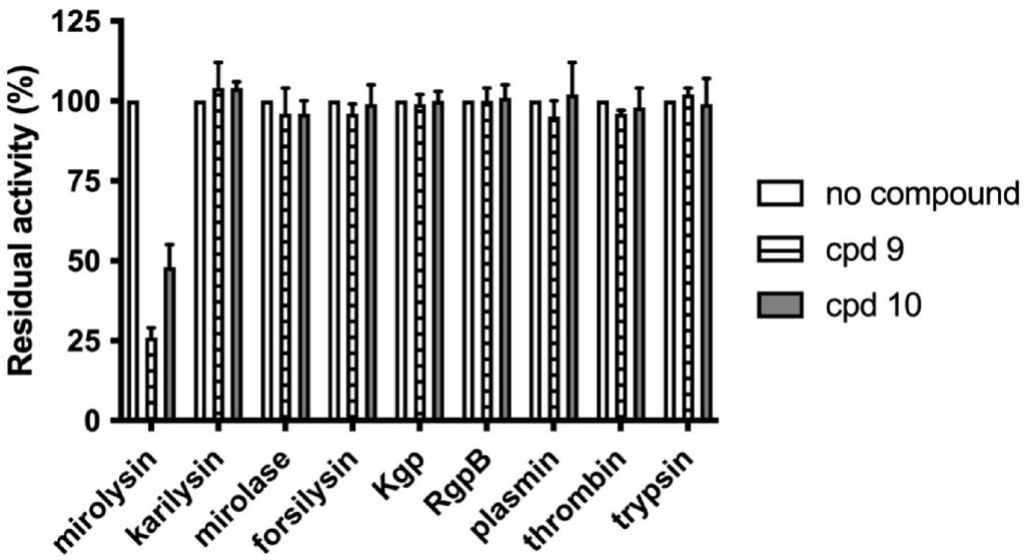
Determination of the specificity of mirolysin inhibitors. Different bacterial and mammalian proteases: KLIKK proteases of *T. forsythia* (mirolysin, karilysin, mirolase, and forsilysin), *P. gingivalis* gingipains (Kgp and RgpB), human thrombin and plasmin, and bovine trypsin were preincubated with a 100 M excess of cpd **9** and cpd **10**, and residual activity was determined using Azocoll or FTC-casein (gingipains). The activity of each enzyme alone was arbitrarily taken as 100%. Results presented are mean ± SD (*N* = 3).

To determine the selectivity of the interaction between cpd **9** and cpd **10** and mirolysin, inhibition of other KLIKK proteases of *T. forsythia* (mirolase, karilysin, and forsilysin), and proteases hydrolysing the peptide bond containing arginine or lysine (Arg- and Lys-specific gingipains) from *P. gingivalis* (RgpB and Kgp, respectively), human thrombin, and plasmin and bovine trypsin were tested. The presence of cpd **9** and cpd **10** had an impact only on mirolysin, resulting in a decrease in activity by 50% and 20%, respectively. The activity of all other proteases was almost completely unaffected (Figure 8). These data indicate that cpd **9** and cpd **10** have high specificity towards mirolysin.

### Crystal structure of the mirolysin–cpd 9 inhibitory complex

**Figure 9.**
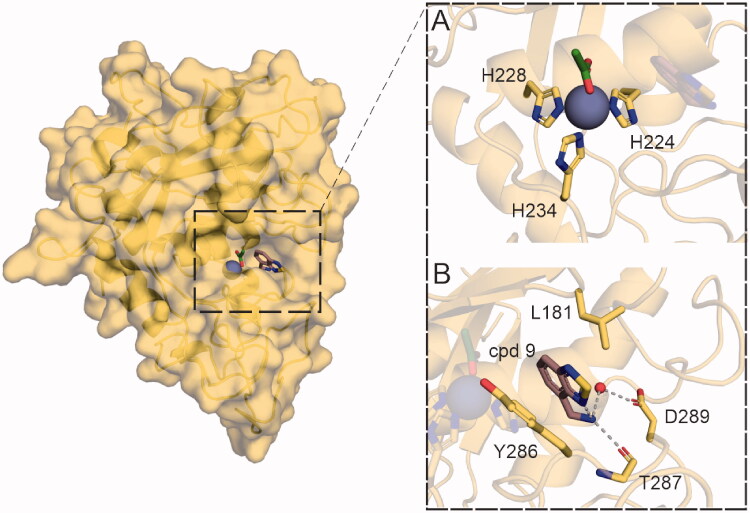
Crystal structure of the mirolysin–cpd **9** complex. Left panel shows the overall structure of mirolysin with bound cpd **9**. (A) Zn ion (grey sphere) coordinated by His224, His228, and His234, with an acetate anion (green) located on top of the zinc ion. (B) Interactions of mirolysin residues and cpd **9**. Hydrogen bonds are depicted as green dashed lines.

As identified by the enzymatic assays described above, cpd **9** is a selective and competitive inhibitor of mirolysin. To determine the molecular mechanism of the interaction of cpd **9** with mirolysin, the crystal structure of the inhibitory complex was solved (Figure 9). The obtained data allowed us to solve the structure in the P1 space group, representing six protein molecules in the asymmetric unit. All molecules show evidence of bound cpd **9**, represented by electron density in the difference electron-density map (*F*o – *F*c) close to the active site of the enzyme (Supplementary Figure 6). This site is occupied by substrate residues in the structure of mirolysin bound to cleaved peptide^20^ (PDB accession no. 6R7W) (Supplementary Figure 7). Although the coverage of the fragments by electron density differs for the different protein molecules in the presented structure, unambiguous placement of cpd **9** was achieved for each protein molecule in the unit cell.

Fragment location highly correlates with the position of the positively charged residues present in both promirolysin and substrate-bound structures (PDB accession nos. 6R7V^20^ and 6R7W^20^, respectively (Figure 9(A,B))). Cpd 9 is inserted deep in the S1′ subsite of the substrate-binding pocket formed by Asp94, Leu181, Tyr216, Arg220, Thr221, His224, Tyr286, Thr287, and Met292. Interactions between cpd **9** and S1′ subsite wall-forming residues are well resolved. The main binding contributions come from a hydrogen bond between the backbone carbonyl of Thr287 and the primary amine of cpd **9**, water-mediated interaction of the cpd **9** primary amine and the Asp289 side chain, and *π*–*π* stacking interactions of the Tyr286 side chain and the ring system of the fragment, as well as *π*–alkyl interaction of Leu181 with the same moiety of cpd **9** (Figure 9(B)).

## Discussion

Peptidases (proteases) are widely distributed among prokaryotes and constitute approximately 3% of all expressed putative proteins. However, the total number of genes encoding peptidases in prokaryotic genomes differs greatly, ranging from only a few in bacterial species in the genus *Mycoplasma* to up to 179 in *Bacillus cereus*^47^. Therefore, because of this wide distribution, it is not surprising that bacterial proteases are involved in processes, such as hydrolysis of extracellular polypeptides to generate nutrients, degradation of misfolded proteins, regulation of gene expression, and post-translational modification of proteins. However, proteolysis is irreversible; thus the activity of secretory proteases in Gram-negative bacteria must be tightly controlled in both a spatial and temporal manner. The first is achieved by compartmentalisation of synthesised proteins, which are directed to the periplasm or extracellular milieu by different secretory pathways. In the case of promirolysin, the N-terminal SP directs the protein to the general secretion system SecYEG. During translocation through the inner membrane, the SP is cleaved off, and promirolysin folds in the periplasm. Similar to other KLIKK proteases of *T. forsythia* and gingipains (proteolytic enzymes of another periodontopathogen, *P. gingivalis*), mirolysin is equipped with a second sorting signal,a conserved CTD recognised by the recently characterised T9SS. After folding in the periplasm, a CTD directs proteins for translocation through the outer membrane by T9SS. Finally, the CTD is cleaved off by PorU sortase, and a cargo protease is either released to the extracellular milieu (*T. forsythia*) or covalently attached to the bacterial surface through anchoring to anionic lipopolysaccharide (*P. gingivalis*)^48^.

During the journey through both membranes, mirolysin is folded and temporarily present in the periplasm. To prevent inappropriate proteolytic damage to any periplasmic proteins, mirolysin, like many other proteases, including gingipains^23^, is maintained as an inactive zymogen by the NTP. The NTP has to be removed to generate a fully active proteolytic enzyme. Such a scenario was described for promirolysin, proM^wt^, for which the NTP was proteolytically removed through cleavage at position S54R and further degraded into small peptides. This event led to the generation of mature mirolysin consisting of only the CD (Figure 2(A,B)). However, the level of latency provided by the mirolysin NTP is extremely high. During proM^wt^ autoprocessing, initial activity was detected after 8 h, and ∼2 weeks was necessary for the completion of the maturation process (Figure 2(A,B)). This is in stark contrast to other proteases, such as another KLIKK protease, karilysin (5 min was sufficient to remove the NTP, and 8 h was sufficient to reach maximum activity^19^, and ulilysin, a close homolog from *Methanosarcina acetivorans* (10 h to complete autoprocessing)^49^. The promirolase NTP, on the other hand, is removed within 30 min but remains noncovalently associated with the CD, inhibiting its proteolytic activity for an additional 48 h^18^. Such a high level of promirolysin latency will definitely prevent autoactivation of the zymogen in the periplasm during the two-step secretion process but seems to be less desirable when the protein is released into the extracellular milieu at the site of infection in human dental pockets. Therefore, it is tempting to speculate that *in vivo* mirolysin processing is accelerated by an external agent such as, for example, another protease or cofactor. However, verification of this very attractive hypothesis is the subject of future work.

Mutation of Cys23 in the mirolysin NTP into both Ala and Leu (proM^C23A^ and proM^C23L^) resulted in almost complete loss of zymogenicity, and the time needed for removal of the NTP shortened from several days to 5 min (Figure 2(A,B)). This finding corroborates previous biochemical and structural studies: a lack of latency is observed for promirolysin expressed with an alternative SP, lacking five amino acids, including Cys23^17^, while the crystal structure of promirolysin indicates that Cys23 interacts with the catalytic zinc^20^. Taken together, all these results confirmed the essential role of Cys23 in mirolysin latency. Thus, mirolysin zymogenicity is achieved by a “cysteine-switch” or Velcro mechanism, best described for human mammalian matrix metalloproteinases (MMPs)^20^. In this mechanism, the fourth coordination spot of catalytic zinc in the zymogen is occupied by the thiol group of a cysteine side chain, thus blocking access of catalytic water to the zinc. The zymogenicity of karilysin, a bacterial MMP, is achieved not by cysteine but by aspartic acid (“aspartate switch”)^50^. On the other hand, as for karilysin, the mirolysin NTP could be removed through autoprocessing, while mammalian MMPs require external proteases, such as trypsin, plasmin, and other MMPs, for activation^20^. During *in cis* activation (Figure 2(A,B)) of proM^C23A^ and proM^C23L^, cleavage of the NTP is immediately followed by further degradation of the protein into small peptides, and only a small fraction of zymogen matured into the active stable proteolytic enzyme. This suggests that Cys23 is responsible not only for latency but also for the stability of the zymogen. This hypothesis was confirmed by activation *in trans*, in which proM^E225A^ was almost resistant to proteolysis (activation) by mirolysin, while both mutant proteins, proM^E225A/C23A^ and proM^E225A/C23L^, were processed into the mature form. As in activation *in cis*, only a small fraction of both mutant proteins were not completely degraded into small fragments and remained as a stable 31 kDa form (Figure 2(D)).

The role of Cys23 in the stability of promirolysin was finally confirmed by differential scanning fluorimetry, showing that replacing Cys decreased the thermal stability of proM^wt^ (Figure 1(B)). A similar role of the NTP in stabilisation of the zymogen was also described for karilysin^50^. However, in stark contrast to karilysin, removal of the NTP did not decrease thermal stability but dramatically increased thermal stability by >30 °C (Figure 1(B)). Thus, it is not surprising that mirolysin efficiently degrades proteinaceous substrates even at 75 °C (Figure 1(E)). Mirolysin’s specificity, cleaving before basic amino acids, not after, as in the case of trypsin, which simplifies analysis of spectra, and its very high thermophilicity, which aids degradation of substrates, suggest that this enzyme may be useful in mass spectrometric analysis for generation of peptides. Such application of enzymes with dual N-terminal specificity towards basic amino acids was described for ulilysin from *M. acetivorans*^51^ and Tryp-N from a thermophilic filamentous fungus, *Chaetomium thermophilum*^52^.

Through NMR-based screening of a fragment library, we identified two compounds, cpd **9** and cpd **10**, which are specific and reversible inhibitors of mirolysin. Of these two compounds, cpd **9** is a much better inhibitor, not only because of its sevenfold lower *K*_i_ value but also because of its ability to inhibit the activation of proM^wt^ (Figure 4). Detailed analysis of mirolysin inhibition revealed that both compounds are competitive inhibitors of mirolysin (Figure 7). Therefore, both compounds should reversibly bind to the same site as the substrate within the catalytic cleft of mirolysin. This assumption was confirmed experimentally by the solved crystal structure of the mirolysin–cpd **9** complex, in which cpd **9** binds to the S1′ subsite in the substrate-binding pocket, which is responsible for mirolysin’s unique specificity^20^. Unfortunately, we were not able to solve the crystal structure of the mirolysin–cpd **10** complex. As a result, both compounds were docked into the mirolysin–product complex crystal structure (PDB accession no. 6R7W)^20^. Bound peptide and the citric acid molecule bound to the catalytic zinc were removed from the active site of the enzyme’s structure prior to the docking procedure using AutoDock 4.2^37^ (Supplementary Figures 8,9). The correctness of the performed docking procedures is demonstrated by the fact that, in both the solved structure and the structure predicted by docking of the mirolysin–cpd **9** complex (Supplementary Figure 8), cpd **9** is located in the same place (S1′) and in an almost identical orientation in which the aromatic ring system is rotated 180 degrees (comparing docking and X-ray structure poses), creating hydrophobic interactions with Leu181 and Tyr286. In the docking results, both cpds **9** and **10** are found in a deep S1′ subsite within the substrate-binding pocket on the surface of mirolysin adjacent to the mono-Zn^2+^-binding site. The primary amine of cpd **9** forms a hydrogen bond with a negatively charged patch in the proposed binding site, consisting of Glu289. The furan ring of cpd **10** is directed towards Thr221 (Supplementary Figure 8). For cpd **9**, the different clusters show alternative conformations of benzothiadiazole, suggesting that this does not make a significant stabilising interaction (for a full description of docking results, please see supplemental information). Of note, the difference in the interaction of cpd **9** and cpd **10** with mirolysin in the docked structures did not provide a clear explanation on a molecular level for the fact that only cpd **9** inhibited proM^wt^ activation *in cis* (Figure 4). However, docking results showed that the lowest energy, highest population cluster for cpd **10** was not situated deep in the S1′ subsite. By contrast, the two lowest clusters for cpd **9** are situated deep in the binding pocket (Supplementary Figure 9). It could be speculated that differences in the positioning of these two compounds in the binding pocket are responsible for the better inhibitory properties of cpd **9**.

Cpd **9** is a very specific inhibitor of mirolysin. Cpd **9** did not affect the activity of other KLIKK proteases, karilysin, mirolase, and forsilysin, or that of other proteases hydrolysing the peptide bond containing lysine or arginine: gingipains Kgp and RgpB from *P. gingivalis*, bovine trypsin and human plasmin, and thrombin (Figure 8). In contrast to mirolysin, other KLIKK proteases have a strong preference for hydrophobic amino acids at scissile peptide bonds^18^^,^^19^ (forsilysin: based on similarity to thermolysin and other M4 proteases^53^). Thus, the chemical nature of their subsites within the substrate-binding pocket will be unable to accommodate the amine group from cpd **9** or lysine and arginine. Mirolysin and other analysed Arg- or Lys-specific proteases have similar specificity, but they differ by the position of these two amino acids in the scissile peptide bond (P1–P1′): in mirolysin, Arg and Lys are localised at the carbonyl side (P1′ residues) of the peptide bond, and, in other proteases, they are localised at the amino side (P1 residue). However, in stark contrast to Arg and Lys side chains, cpd **9** does not contain an aliphatic straight-chain, aside from the amine group, but instead contains an aromatic ring, which fits well into the deep and spacious S1′ subsite in the mirolysin substrate-binding pocket^20^. This assumption was confirmed by computational docking and subsequently by the solved crystal structure of the mirolysin–cpd 9 inhibitory complexes. As in the case of the product complex of mature mirolysin, Lys at P1′^20^, the amino group of cpd **9** interacts with Thr287 and Asp289 at the bottom of the S1′ pocket, but additionally, the aromatic ring of cpd **9** interacts with Leu181 and Tyr286 in the walls forming the S1′ subsite. This location fits well with our predictions, explaining the specificity of cpd **9**, for which the amino group mimics the positively charged amino group of the Lys side chain, and the bulky ring system fills the S1′ pocket, providing hydrophobic contacts with the environment in a manner similar to the aliphatic part of the Lys side chain of peptides^20^ (Supplementary Figure 7). Other interactions reported in the mirolysin–product complex structure^20^ are not present in the mirolysin–cpd **9** complexes because of the smaller size and different chemical structure of the ligand present on top of the zinc ion (citrate and acetate in the peptide^20^ – and compound-bound structures, respectively).

Interaction of cpd **9** with mirolysin was described by a few parameters: a *K*_D_ of 72 and 1.1 µM, determined by NMR titration of ^15^N-labelled mirolysin and MST, respectively; an IC_50_ of 2.3 µM; and a *K*_i_ of 3.2 µM. So far, several studies were reported that employed NMR-based screening to identify therapeutic compounds or described development of inhibitors of secretory proteases of human periodontopathogens from the “red complex” of bacteria. In one of the studies, NMR screening was used to identify antagonists of p53–Mdm2 interactions, which play an important role in carcinogenesis. Identified compounds bound to Mdm2 with *K*_D_ values ranging from 1 to 365 µM and from 0.5 to 40.5 µM, which were determined by NMR titration of ^15^N-labelled Mdm2 with compounds and MST, respectively. As in this study, large differences were also observed between *K*_D_ values determined by NMR titration and MST^54^. In the other case, phage display was employed to identify peptide inhibitors of another KLIKK protease, karilysin. The identified peptides could be shortened to the tetrapeptide SWFP and inhibited karilysin with a *K*_i_ ranging from 5 to 11 µM^24^. Compared with both of these studies, cpd **9** exerted similar affinity or inhibitory potency towards mirolysin. Considering the well-described role of *P. gingivalis* and its secretory proteases in the aetiology of periodontitis, it is not a surprise that gingipains were a target for the development of potent and specific inhibitors. Among others, inhibitors of Arg-specific gingipains, Rgp (Kyt-1 (*K*_i_ = 130 pM)^22^ and COR286 (IC_50_ ≤ 50 pM)^7^), and the Lys-specific gingipain, Kgp (Kyt-36 (*K*_i_ = 75 pM)^22^ and COR217 and COR119 (IC_50_ ≤ 50 pM)^7^), are much more potent than cpd **9**. In contrast to cpd **9**, all gingipain inhibitors possessed not only lysine or arginine interacting with the S1 subsite responsible for the proteases’ substrate specificity, but also other fragments attached to both termini of Lys and Arg, which could bind to other subsites within the catalytic cleft and, as a result, increase the overall affinity of the inhibitor binding to gingipains. This hypothesis was experimentally verified by solving the crystal structure of the Kgp–Kyt-36 inhibitory complex, in which Kyt-36 tightly covered subsites S3–S1′ in the catalytic cleft of the enzyme^55^. Thus, in future work, the inhibitory properties of cpd **9** could be enhanced by a fragment-growing approach through rounds of medicinal chemistry to maximally exploit the possible interactions in the S1′ pocket and beyond. One of the possibilities would employ additional positively charged moieties targeting the Tyr286 backbone carbonyl and the side chain of Glu260, creating opportunities for additionally charged interactions outside the S1′ pocket, and possibly creating an extra exit vector for improving the solubility of the compound at the solvent-exposed part. Furthermore, a parallel approach of fragment growing in the direction of the zinc ion would provide the potential for further interactions. Several moieties, including hydroxamate, thiolate, or carboxylate, offer a strong affinity towards zinc ions and could thus be included in developing the cpd **9** structure, which would greatly increase the binding affinity as has been shown before for other molecules targeting zinc-containing enzymes^56^. Applying the strategy based on growing the fragment in both directions would result in better enzymatic inhibitory properties, together with higher binding affinity of the compound.

In conclusion, our findings not only confirmed that mirolysin latency is achieved by the “cysteine-switch” mechanism but also significantly improved our knowledge about this secretory protease from *T. forsythia*. First, mirolysin zymogenicity is exerted by Cys23 in the NTP, which provides latency for promirolysin for several days. Mutation of Cys23 also decreases thermal stability and resistance to proteolysis of the protein. Second, mirolysin possesses extreme thermophilicity, reaching its optimal activity at 65 °C. Third, through NMR-based screening of a fragment library, we identified cpd **9**, which is a very selective and competitive inhibitor of mirolysin. Cpd **9** inhibits mirolysin activity with a *K*_i_ of 3.2 µM and maturation of promirolysin into an active protease. The solved crystal structure reveals that cpd **9** binds to the S1′ subsite of the substrate-binding pocket in a manner similar to the peptide in the mirolysin–product complex. Based on the solved structure of the mirolysin–cpd **9** complex, in the future, the inhibitory potency of cpd **9** could be improved by fragment growing. Such a compound, together with inhibitors against the most relevant proteases secreted by periodontopathogens, may be used not only in the treatment but also in the prevention of periodontitis. Compounds of such a preventative character could be added to existing oral care products such as toothpaste, mouthwash, or even chewing gum.

## Supplementary Material

Supplemental Material
