# Supplementary material for: Latency, thermal stability, and identification of an inhibitory compound of mirolysin, a secretory protease of the human periodontopathogen Tannerella forsythia
Source: J Enzyme Inhib Med Chem. 2021 Jul 1;36(1):1267–81. doi: 10.1080/14756366.2021.1937619 (PMC8259862; doi:10.1080/14756366.2021.1937619)
Supplement: Supplemental Material [file IENZ_A_1937619_SM3682.pdf]

Latency, thermal stability, and identification of inhibitory compound of mirolysin, a secretory protease of human periodontopathogen *Tannerella forsythia.*

Krzysztof M. Zak, Mark J. Bostock, Irena Waligorska, Ida B. Thøgersen, Jan J. Enghild, Grzegorz M. Popowicz, Przemyslaw Grudnik, Jan Potempa, Miroslaw Ksiazek

Supplementary materials

Supplementary methods

Chemical shift perturbation (CSP) analyser

^{1}H,^{15}N HSQC spectra were evaluated using the CSP Analyser [1]. Peaks were picked using the TopSpin peak-picking function and imported to the CSP analyser. To reduce the number of peaks for the CSP Analyser to below the limit of 150, a cut-off of 8.3 ppm was used, excluding peaks in the crowded centre of the spectrum and the asparagine and glutamine side-chains.

Screening of inhibitory properties

Mirolysin (40 nM) was mixed with each of the 21 compounds (cpds) (2 µM) in assay buffer (50 mM Tris, 150 mM NaCl, 5 mM $CaCl_2$, 0.02% NaN_3, 0.05% Pluronic F-127, pH 7.5) in a 96-well plate (total volume 100 µl). After 15 min at 37 °C, 100 µl of the substrate solution, FTC-casein (ThermoFisher Scientific, Waltham, MA, USA) (100 µg/ml), was added. Substrate hydrolysis was monitored for 60 min at 37 °C by measuring fluorescence (ex/em = 485/538 nm) with a SpectraMax Gemini XS microplate reader (Molecular Devices, San Jose, CA, USA). The inhibition percentage was determined regarding the enzyme's sample without any additives: cpd or DMSO (control sample).

Supplementary results

Fragment docking

Two fragments (cpds 9 and 10) were docked into the mirolysin crystal structure (PDB accession no: 6R7W) [2]. Bound peptide and citric acid molecules were removed from the

active site of the enzyme's structure before the docking procedure using Autodock 4.2 with AutoDockTools [3].

Both compounds are found in a deep cleft on the surface of mirolysin adjacent to the mono-Zn^{2+} binding site. Clustering of the docked compounds shows clear differences in energy and population: for cpd 10, the lowest energy conformation shows the highest population; for cpd 9, three clusters are at lower energy than the highest population cluster. The crystal structure of mirolysin [2] shows a citric acid molecule interacting with the entrance to the same pocket. Cpds 9 and 10 contain a primary amine, which forms a hydrogen bond with a negatively charged patch in the proposed binding site; for the lowest energy docked conformation of cpd 9, this points towards Thr^{287} and Asp^{289}; for the lowest energy docked conformation of cpd 10, the primary amine points 180 degrees in the opposite direction towards catalytic Glu^{225}. The lowest energy docked poses for the four lowest energy clusters are shown in Supplementary Figure 9. For cpd 9, the different clusters show alternative conformations of the benzothiadiazole, suggesting this functional group does not make a significant stabilising interaction. For cpd 10, the two lowest energy clusters, which consist of >90% of the confirmations, show deviations in the orientation and position of cpd 10 in the binding pocket. The three lowest energy clusters are also close in energy (-7.83 to -7.73 kcal/mol), suggesting there is not a strong preference for the position of cpd 10 in the binding pocket (Supplementary Figure 9).

Supplementary references

1) Fino R, Byrne R, Softley CA, Sattler M, Schneider G, Popowicz GM. Introducing the CSP Analyzer: A novel Machine Learning-based application for automated analysis of two-dimensional NMR spectra in NMR fragment-based screening. Comput Struct Biotechnol J. 2020;18:603-611.
2) Guevara T, Rodriguez-Banqueri A, Ksiazek M, Potempa J, Gomis-Rüth FX. Structure-based mechanism of cysteine-switch latency and of catalysis by pappalysin-family metallopeptidases. IUCrJ. 2020;7(Pt 1):18-29.
3) Morris GM, Huey R, Lindstrom W, Sanner MF, Belew RK, Goodsell DS, Olson AJ. AutoDock4 and AutoDockTools4: Automated docking with selective receptor flexibility. J Comput Chem. 2009;30(16):2785-91.

Tables

Supplementary Table 1. Chemical formulas of tested compounds, the qualitative magnitude of chemical shift perturbations (range 0-5) observed in $^{1}H,^{15}N$ HSQC experiments, CSP Analyser scores[1] and enzyme activity measurements (%).

Fragment number	Fragment formula	Magnitude of chemical shift perturbations	CSP Analyser probability score	Residual activity (%)
1	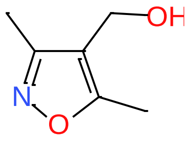	1	0	93
2	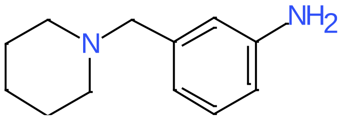	0	0	86
3	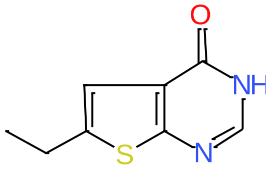	0	0	96
4	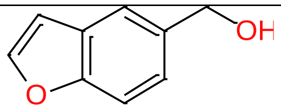	0	0	84
5	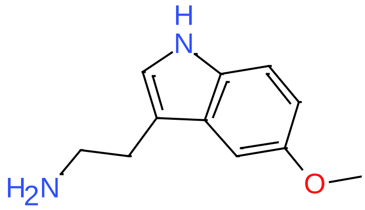	1	0	78
6	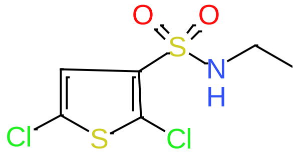	1	0	77
7	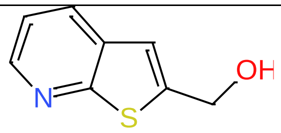	0	0	95

8	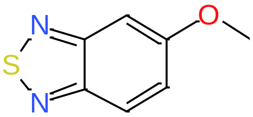	2	0	100
9	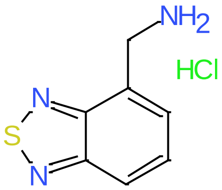	5	0.11	86
10	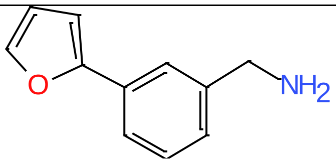	4	0.01	84
11	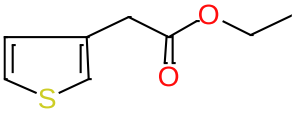	1	0	84
12	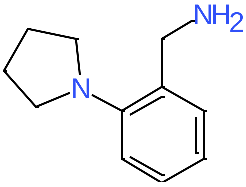	0	0	78
13	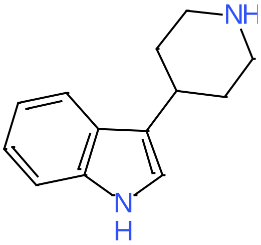	2	0	94
14	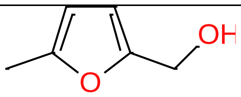	0	0	87
15	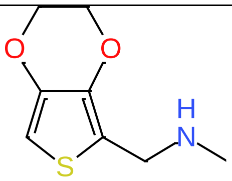	1	0	95
16	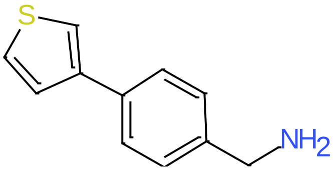	1	0	97

17	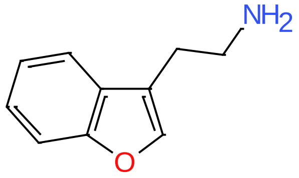	4	0.95	99
18	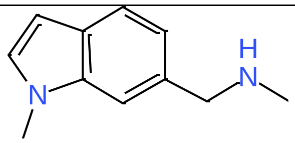	2	0	95
19	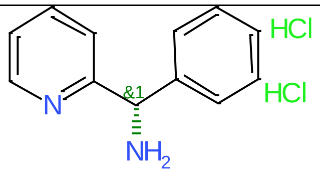 racemate	0	0	96
20	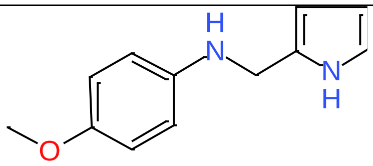	1	0	96
21	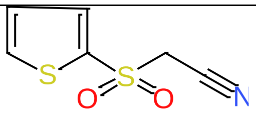	0	0	100

Supplementary Table 2. Data collection and refinement statistics for the mirolysin-cpd 9 inhibitory complex (PDB accession no: 7OD0).
Values in parentheses represent the highest-resolution shell.

Data collection	
Wavelength (Å)	0.99
Space group	P 1
Cell dimensions	
a, b, c (Å)	77.59, 77.64, 81.99
α, β, γ (°)	99.46, 90.00, 119.98
Resolution range (Å)	38.80 - 2.10 (2.14 - 2.10)
Total reflections	228487 (10680)
Unique reflections	88998 (4272)
Mean I/sigma(I)	7.80 (3.40)
Completeness (%)	92.20 (87.20)
Multiplicity	2.60 (2.50)
CC1/2	0.98 (0.93)
Wilson B-factor (Å2)	10.40
**Refinement**	
R-work	0.20
R-free	0.23
Number of atoms	
non-hydrogen atoms	14258
macromolecules	13051
ligands	143

solvent	1064
RMSD	
bonds (Å)	0.015
angles (°)	1.76
Ramachandran favoured (%)	97.18
Ramachandran allowed (%)	1.82
Ramachandran outliers (%)	1.00
Rotamer outliers (%)	2.38
Clashscore	8.17
B-factors	
average	16.84
macromolecules	16.08
ligands	19.56
solvent	25.81

Figures

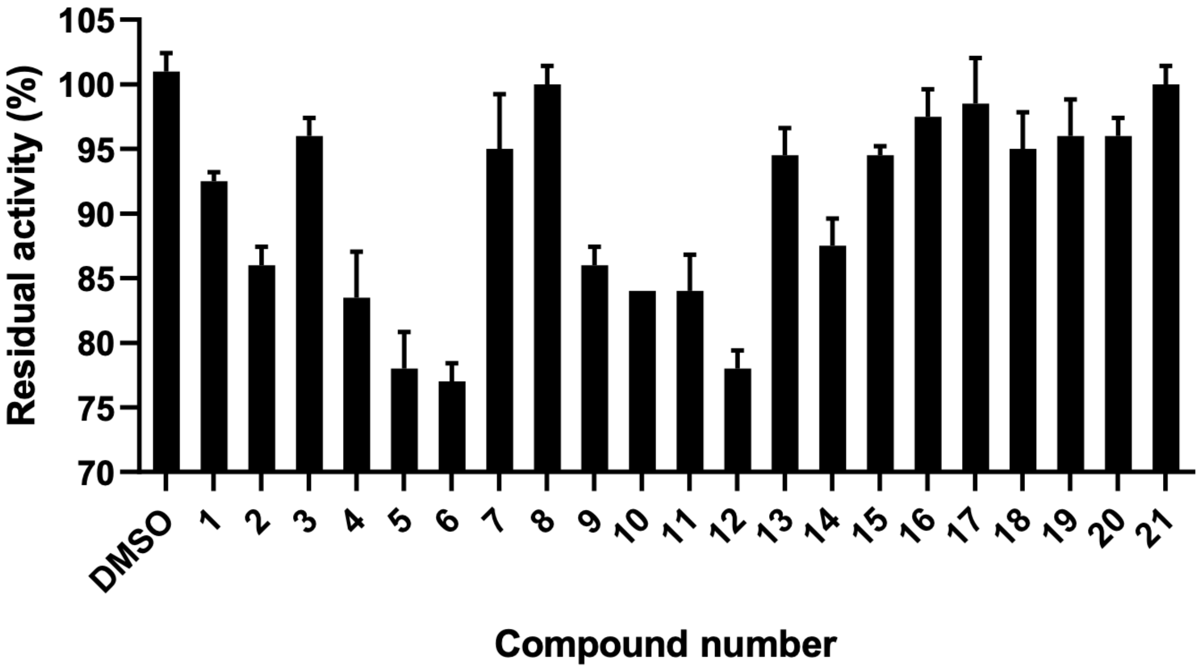

Supplementary Figure 1: Screening round II: inhibitory properties of 21 compounds selected in screening round I against mirolysin.

Mirolysin was incubated with 50 M excess of each of the 21 compounds, and then the residual activity was determined employing FTC-casein as a substrate. The activity of enzyme alone, without cpd or DMSO, was arbitrarily taken as 100%.

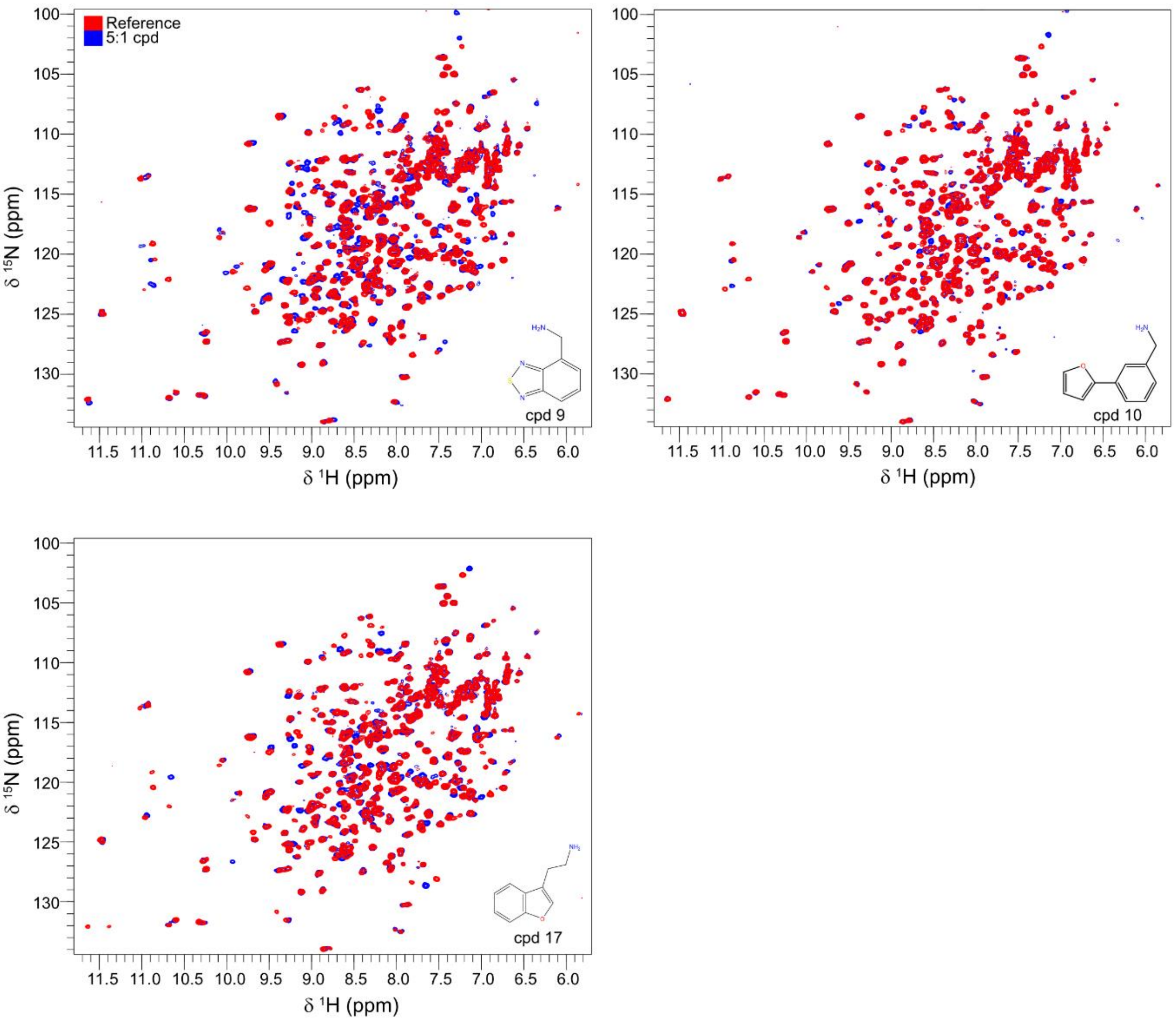

Supplementary Figure 2**.** Fragment validation using 2D [^{1}H,^{15}N]-HSQC spectra.

2D [^{1}H,^{15}N]-HSQC spectra were used to validate the 1D screening results from STD deconvolution screening. Spectra were recorded with ^{15}N-labelled mirolysin and a compound added in a 5:1 ratio. Spectra are shown for the three fragments showing the largest number of chemical shift perturbations. These fragments were also identified by the CSP analyser software[1]. The reference spectrum is shown in red, and spectra with fragment at a 5:1 ratio are shown in blue.

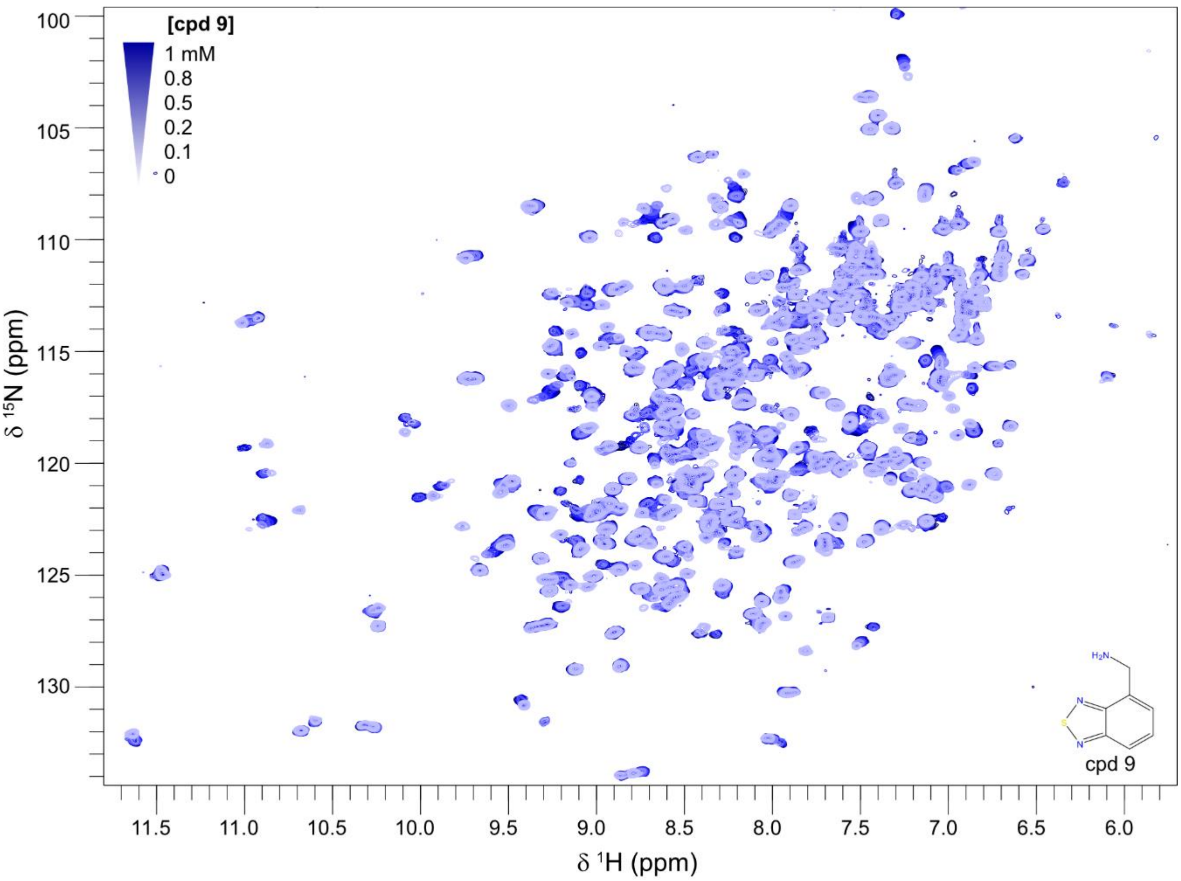

Supplementary Figure 3. NMR titration of cpd 9 against ^{15}N-labelled mirolysin.
2D [^{1}H,^{15}N]-HSQC spectra are shown for titration of cpd 9 from 0 to 1 mM (light blue to dark blue) against ~80 μM ^{15}N-labelled mirolysin. Fitting curves are shown in the main text, Figure 3.

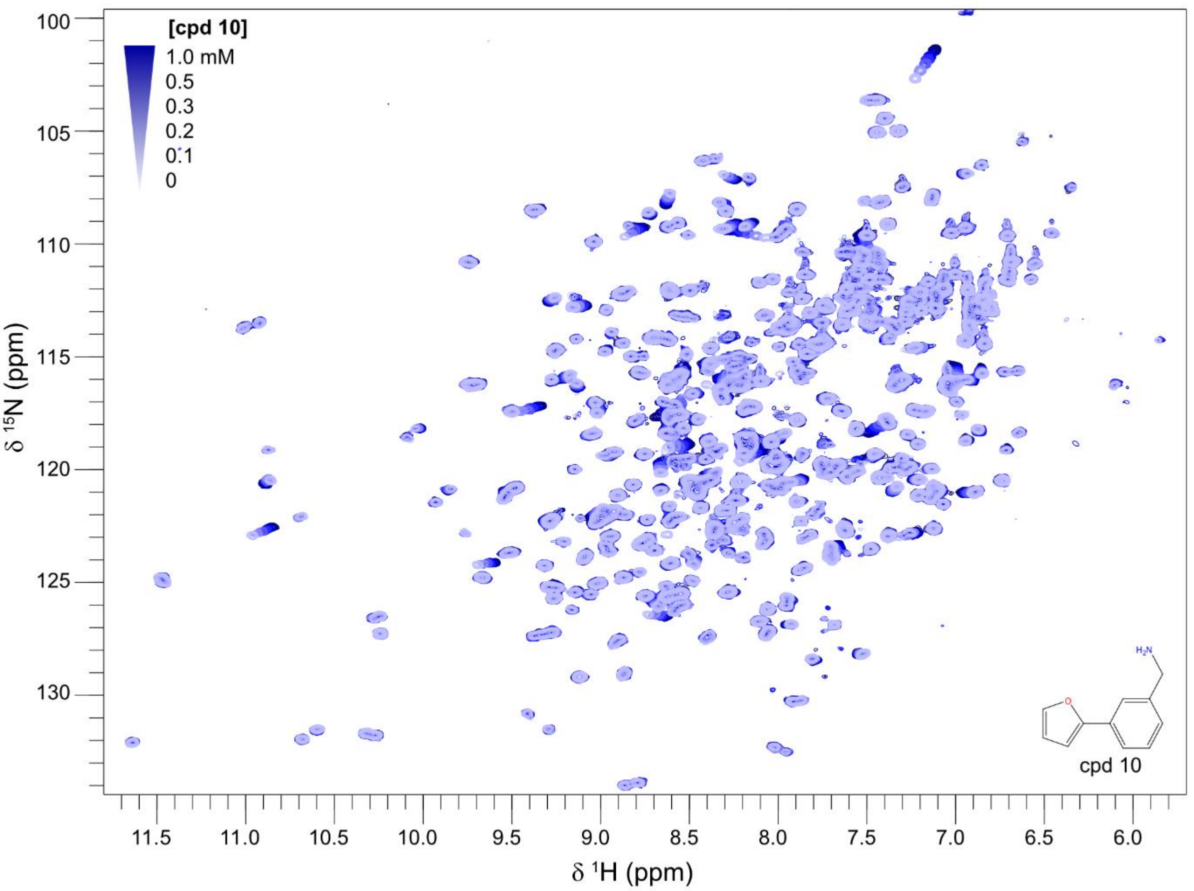

Supplementary Figure 4. NMR titration of cpd 10 against ^{15}N-labelled mirolysin

2D [^{1}H,^{15}N]-HSQC spectra are shown for titration of cpd 10 from 0 to 1 mM (light blue to dark blue) against ~75 μM ^{15}N-labelled mirolysin. Fitting curves are shown in Supplementary Figure 5.

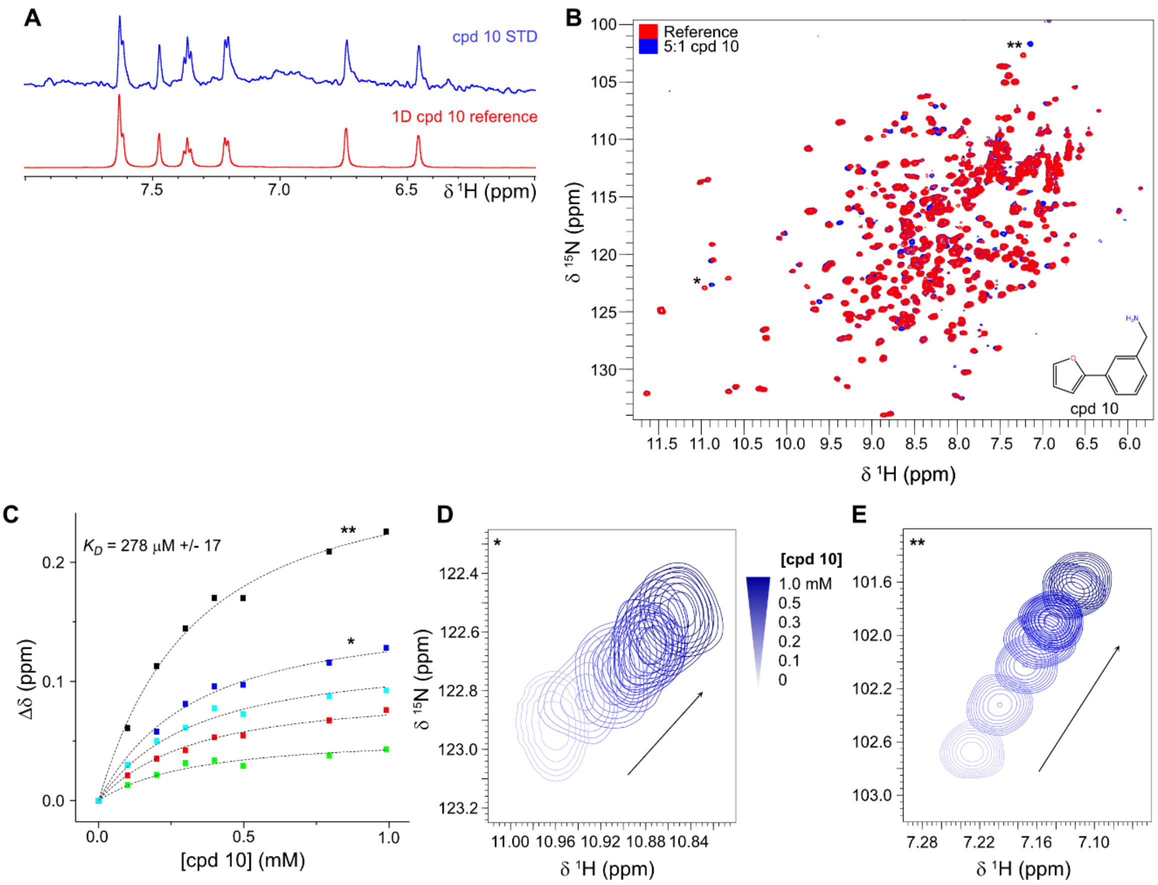

Supplementary Figure 5. NMR analysis of the interaction of cpd 10 with mirolysin.

A) Amide region of an STD spectrum for cpd 10, identified in the STD deconvolution screen. **B)** 2D [^{1}H,^{15}N]-HSQC experiment for ^{15}N-labelled mirolysin showing chemical shift perturbations on the addition of cpd 10, added at a 5:1 ratio (blue) compared with a reference spectrum. **C)** K_D is approximated using a global fit to five residues observed to be in fast exchange. **D)** and **E)** show examples of two peaks in fast exchange on titration of cpd 10 from 0 to 1 mM (light blue to dark blue). The peaks are indicated in B) and C) by * and **, respectively. The full spectra are shown in Supplementary Figure 4.

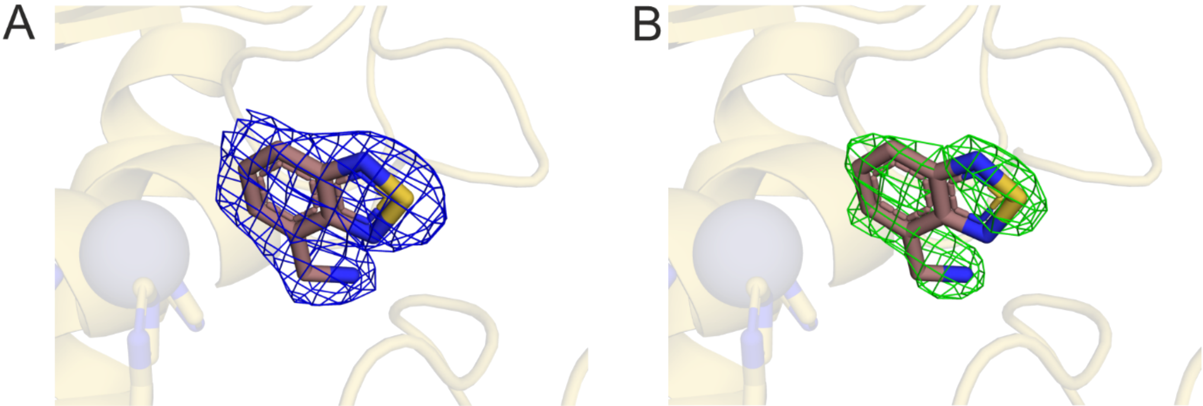

Supplementary Figure 6. Electron density maps of cpd 9 located in chain C of the structure.

A) 2Fo-Fc map contoured at 1.0 σ. B) Fo-Fc omit map contoured at 3.0 σ.

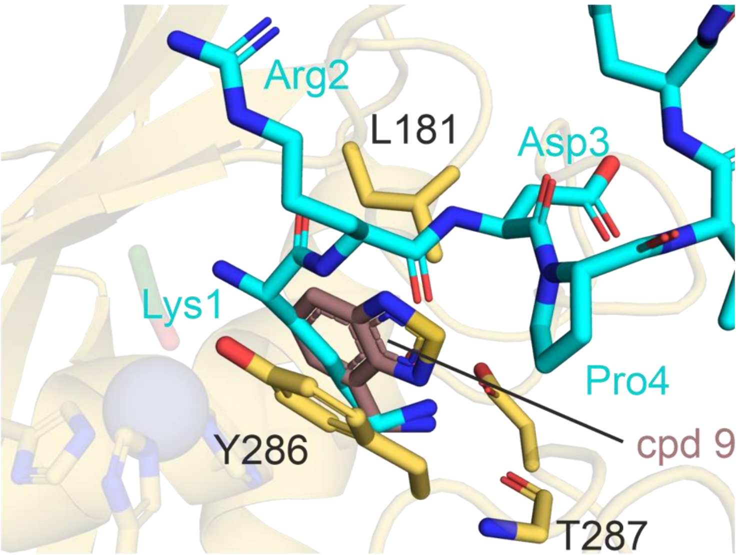

Supplementary Figure 7. Superposition of mirolysin-cpd 9 structure with a peptide derived from the mirolysin-product structure (PDB accession no: 6R7W[2]).

Mirolysin-cpd 9 structure is presented in gold, a peptide derived from the mirolysin-substrate structure is shown in cyan.

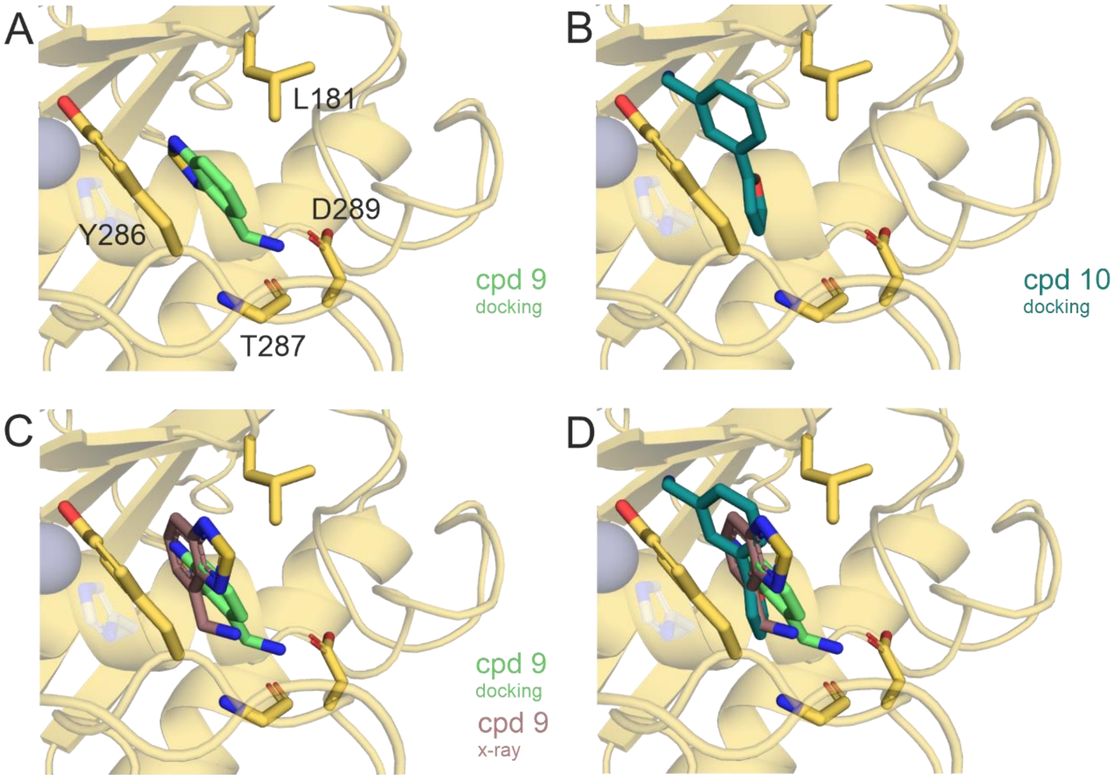

Supplementary Figure 8. Docking results for cpds 9 and 10.

The most energetically favourable conformations are presented for docked cpd 9 and 10. A) Docking pose of cpd 9. B) Docking pose of cpd 10. C) Superposition of poses of cpd 9 from docking and crystal structure of mirolysin-cpd 9 inhibitory complex. D) Superposition of poses of cpd 9 from docking and crystal structure and pose of cpd 10 from docking.

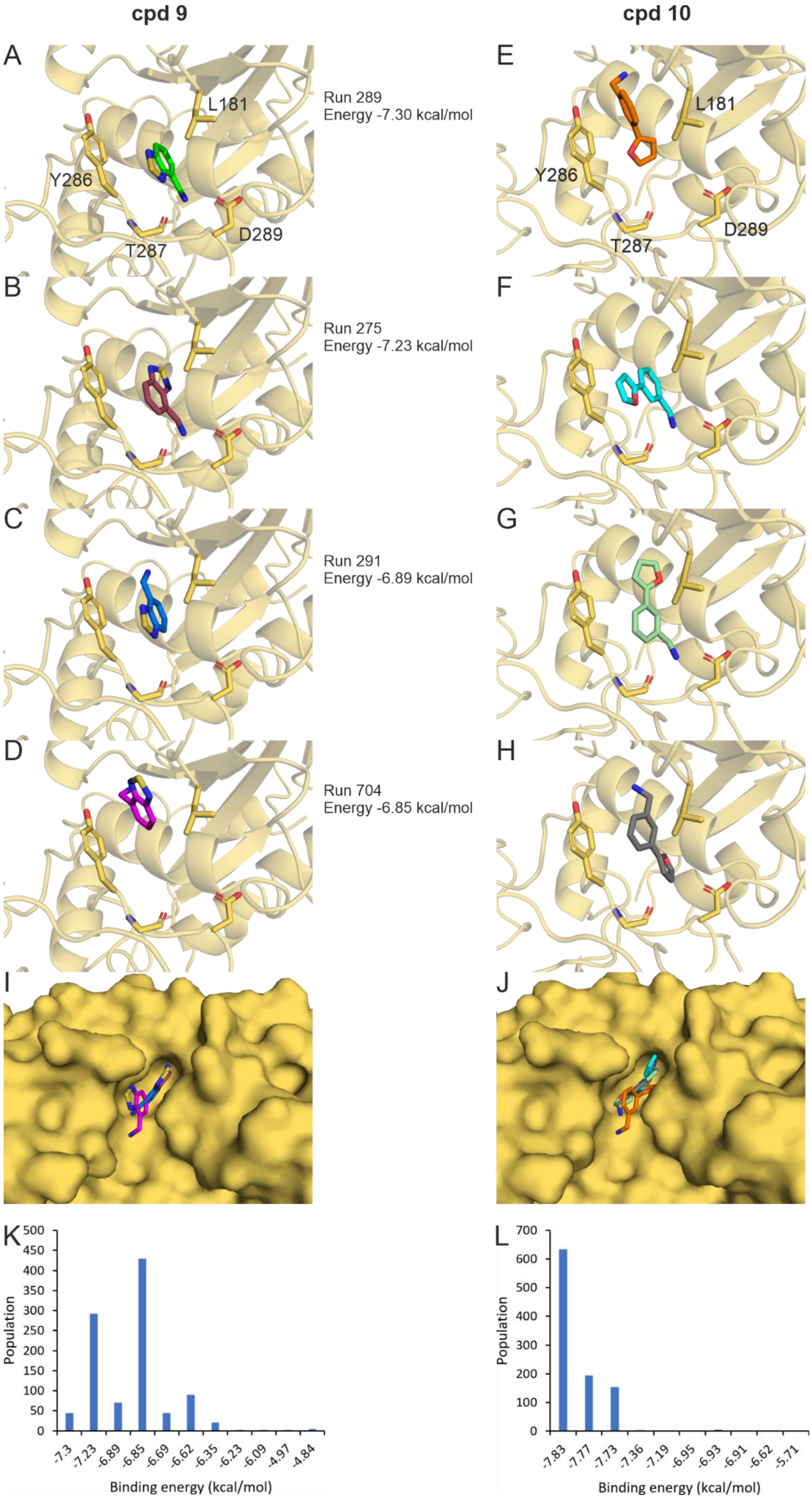
cpd 9
cpd 10
A
L181
Y286
T287
D289
Run 289
Energy -7.30 kcal/mol
E
L181
Y286
T287
D289
Run 860
Energy -7.83 kcal/mol
B
Run 275
Energy -7.23 kcal/mol
F
Run 311
Energy -7.77 kcal/mol
C
Run 291
Energy -6.89 kcal/mol
G
Run 857
Energy -7.73 kcal/mol
D
Run 704
Energy -6.85 kcal/mol
H
Run 704
Energy -7.36 kcal/mol
I
J
K
Population
Binding energy (kcal/mol)
-7.3 -7.23 -6.89 -6.85 -6.69 -6.62 -6.35 -6.23 -6.09 -4.97 -4.84
0 50 100 150 200 250 300 350 400 450 500
L
Population
Binding energy (kcal/mol)
-7.83 -7.77 -7.73 -7.36 -7.19 -6.95 -6.93 -6.91 -6.62 -5.71
0 100 200 300 400 500 600 700

Supplementary Figure 9: Detailed analysis of docking poses of cpds 9 and 10.

The lowest energy docked conformations for the four lowest energy clusters are presented for cpd 9 (A-D) and cpd 10 (E-H). The run number and calculated binding energy are shown on the right-hand side of each pose. Superposition of all four docked conformations in the substrate-binding pocket is presented for cpd 9 (I) and 10 (J) below the individual representations. Histograms show the populations of the docking clusters plotted against the energy of the lowest energy docked conformation in each cluster for cpd 9 (K) and cpd 10 (L).
